# Diabetic Retinopathy and Cognitive Dysfunction in Type 2 Diabetes Mellitus: A Systematic Review and Meta‐Analysis of Epidemiological Associations and Clinical Implications

**DOI:** 10.1155/jdr/1328324

**Published:** 2026-05-17

**Authors:** Ye He, Yining Zhu, Lixiao Yang, Yuzhu Wang, Chuyi Tan, Fangfang Ma, Ying Xia

**Affiliations:** ^1^ Cardiac Care Unit, Department of Internal Medicine, Peking Union Medical College Hospital, Chinese Academy of Medical Sciences and Peking Union Medical College, Beijing, China, cacms.ac.cn; ^2^ Department of Internal Medicine, Peking Union Medical College Hospital, Chinese Academy of Medical Sciences and Peking Union Medical College, Beijing, China, cacms.ac.cn

**Keywords:** cognitive impairment, dementia, diabetes mellitus, diabetic retinopathy, mild cognitive impairment

## Abstract

**Aims:**

Type 2 diabetes mellitus (T2DM) is linked to both diabetic retinopathy (DR) and cognitive impairment, yet the consistency and modifiers of this relationship remain unclear. This systematic review and meta‐analysis are aimed at quantifying the association between DR and cognitive dysfunction in T2DM and explore variations by cognitive subtype, diabetes duration, follow‐up time, and assessment methods.

**Materials and Methods:**

We searched PubMed, Embase, Web of Science, and Chinese databases from 2000 to March 2025. Observational studies comparing cognitive outcomes in T2DM patients with and without DR were included. Data were pooled using random‐effects models.

**Results:**

Forty‐eight studies were included. Cross‐sectional studies showed a strong association (OR = 2.04, 95% CI: 1.72–2.42; *I* ^2^ = 0.0*%*), whereas cohort studies showed a modest but significant association (OR = 1.13, 95% CI: 1.01–1.26; *I* ^2^ = 85.2*%*). The strongest associations were observed for mild cognitive impairment (cross‐sectional OR = 2.15) and dementia (cross‐sectional OR = 2.20; cohort OR = 1.17). Longer diabetes duration (≥ 10 years) strengthened the association in cross‐sectional studies (OR = 2.05), but not in cohort studies. Extended follow‐up (≥ 10 years) significantly increased the effect in cohort studies (OR = 1.91).

**Conclusion:**

DR is significantly associated with cognitive impairment in T2DM, particularly dementia and its subtypes. The association is influenced by study design, diabetes duration, follow‐up time, and assessment method. DR may serve as a research‐level risk signal for cerebral microvascular damage, warranting further prospective studies to validate its clinical utility.

## 1. Introduction

Type 2 diabetes mellitus (T2DM) constitutes a critical global health challenge, with current estimates indicating approximately 589 million affected adults aged 20–79 years worldwide. Projections suggest this burden will escalate to 853 million by 2050, compounded by significant undertreatment—nearly 60% of individuals lack routine care despite diagnostic confirmation [1–3]. This epidemic drives substantial morbidity from microvascular and macrovascular complications. Among these, diabetic retinopathy (DR) remains a predominant cause of preventable blindness, affecting approximately one‐third of diabetic patients globally. Its insidious onset—frequently asymptomatic until advanced stages—underscores the imperative for systematic screening and early intervention [4, 5].

Concurrently, T2DM independently elevates dementia risk, with meta‐analytic evidence demonstrating a 60% increased incidence compared with nondiabetic populations (RR 1.6, 95% CI 1.5–1.8) [6, 7]. The Lancet Commission identifies diabetes as a modifiable risk factor for dementia, accounting for 1.1% of population‐attributable risk [7]. Mechanistically, microvascular dysfunction is implicated in both DR and cognitive decline. Structural and embryological parallels between retinal and cerebral microvasculature suggest retinal pathology may serve as a noninvasive biomarker for cerebral microangiopathy [8–10]. This is biologically plausible given the retina′s ontogenetic origin as neural tissue and its shared susceptibility to diabetic microvascular injury.

Emerging evidence posits DR as a potential indicator of accelerated cognitive impairment in T2DM, possibly reflecting systemic microvascular compromise [11, 12]. Crucially, there has been no comprehensive meta‐analysis to quantitatively integrate the epidemiological association between DR and cognitive impairment. Previous reviews [13, 14] failed to distinguish between different subtypes of cognitive impairment (such as mild cognitive impairment [MCI], Alzheimer′s disease [AD], and vascular dementia [VD]), even though these subtypes may have different underlying pathophysiological mechanisms and varying associations with microvascular disease. Furthermore, existing studies have several limitations, including narrow inclusion criteria, the simultaneous inclusion of patients with both Type 1 and Type 2 diabetes, and an early cutoff date that excludes recent large‐scale cohort studies [15].

This systematic review and meta‐analysis aims to quantify the cross‐sectional and longitudinal associations between DR and cognitive impairment in individuals with T2DM. Based on this, stratified analysis and subgroup meta‐analysis will be conducted to assess the strength and specificity of this association across different subtypes of cognitive impairment. This study can provide insights into the clinical significance of risk stratification and early intervention strategies, particularly by examining the moderating effects of key factors on the magnitude and direction of the relationship between DR and cognitive function.

## 2. Materials and Methods

This study was designed, conducted, and reported following the Preferred Reporting Items for Systematic Reviews and Meta‐Analyses [16]. The protocol was registered a priori on PROSPERO (Registration No. CRD420251033104).

### 2.1. Eligibility Criteria

The inclusion criteria were as follows: (a) observational studies, (b) T2DM patients, (c) DR diagnosed by validated methods and graded using standardized systems, and (d) cognitive impairment (e.g., MCI, dementia) as the primary outcome.

Exclusion Criteria were as follows: (a) nonoriginal studies (e.g., reviews, case reports) and (b) non‐English or non‐Chinese publications.

### 2.2. Database Search

Two researchers (He and Zhu) conducted supplementary searching of the Pubmed, Embase, Web of Science, Wanfang data, CBM, CNKI, and VIP databases from 1 January 2000 and before by 31 March 2025, independently using the terms “Diabetic Retinopathy,” “Diabetic Retinopathies,” “DR,” “PDR,” “NPDR,” “Cognitive Dysfunction,” “Cognitive Dysfunctions,” “cognitive impairment,” “cognitive decline,” “cognitive defect,” “dementia,”and “Alzheimer′s disease.” The search strategy is enlisted in detail in Table S1. The references and previously published systematic reviews were searched manually for a supplement.

### 2.3. Screening and Data Extraction

To minimize duplication bias, studies were assessed for overlap based on authors, region, population, and data collection period. Overlap was defined as studies from the same region, involving overlapping populations, and conducted during similar timeframes. In such cases, the higher‐quality study was selected according to the following criteria: (1) priority given to longitudinal (cohort/case–control) over cross‐sectional designs; (2) preference for studies using standardized diagnostic tools (e.g., ETDRS for DR, combined MoCA/MMSE for cognition); (3) higher quality scores (Newcastle–Ottawa Scale [NOS] ≥ 7 or Agency for Healthcare Research and Quality [AHRQ] ≥ 8); (4) larger sample size and more complete outcome data. If consensus was not reached, final selection was determined through team discussion. The following data were extracted from each included study: authors, country, patient source, age, diabetes duration, age at onset, HbA1c (%), total sample size (male %), group sample sizes (N/C), follow‐up duration (mean ± SD), DR assessment method, cognitive assessment tool, and the odds ratio (OR) with 95% confidence interval (CI) for the DR–cognitive impairment association. Adjusted confounders were also recorded. To minimize selection bias, two investigators independently screened titles and abstracts, followed by full‐text review of potentially eligible articles.

### 2.4. Quality Assessment

Cross‐sectional studies were evaluated using an 11‐item instrument from the AHRQ (https://www.ncbi.nlm.nih.gov/books/NBK35156/), with total scores of 0–3, 4–7, and 8–11 indicating low, moderate, and high quality. Methodological quality was appraised using the NOS [17] for case‐control and cohort studies, which assess selection, comparability, and exposure/outcome ascertainment on a star‐based system (0–9 stars). Studies scoring 0–3, 4–6, and 7–9 stars were rated as low, moderate, and high quality, respectively. Two investigators (He and Zhu) independently conducted the assessments.

### 2.5. Data Analysis

Data extraction was performed using standardized forms to collect effect estimates (ORs or hazard ratios [HRs]) and their 95% CIs. The primary effect measure was the OR. Case–control studies were excluded from the primary meta‐analysis due to higher risk of bias but retained for qualitative synthesis. For studies reporting HRs, we converted them to log(OR) using the approximation log(HR) ≈ log(OR) under the assumption of a low outcome event rate (< 10%) [18].

A random‐effects model (DerSimonian–Laird [DL] method) was used to account for anticipated heterogeneity, which was assessed using the *I* ^2^ statistic (*I* ^2^ > 50*%* indicating substantial heterogeneity) and Cochran′s Q test (*p* < 0.10). To explore sources of heterogeneity, we performed both subgroup analyses and metaregression. Subgroup analyses examined the influence of single categorical moderators (e.g., diabetes duration, follow‐up time, and cognitive assessment tool) by stratifying studies. Meta‐regression was used to assess the independent contribution of multiple study‐level characteristics simultaneously, and the specific covariates included are described in the Results section. To further assess robustness, sensitivity analyses were performed using restricted maximum likelihood (REML) for robustness. Publication bias was evaluated using funnel plots, Egger′s test, and trim‐and‐fill adjustment. All analyses were performed using Stata 18.0, with statistical significance set at two‐tailed *p* < 0.05. Missing data were requested from corresponding authors, and discrepancies in extracted data were resolved through consensus or third‐party arbitration.

## 3. Results

### 3.1. Literature Search

**Figure 1:**
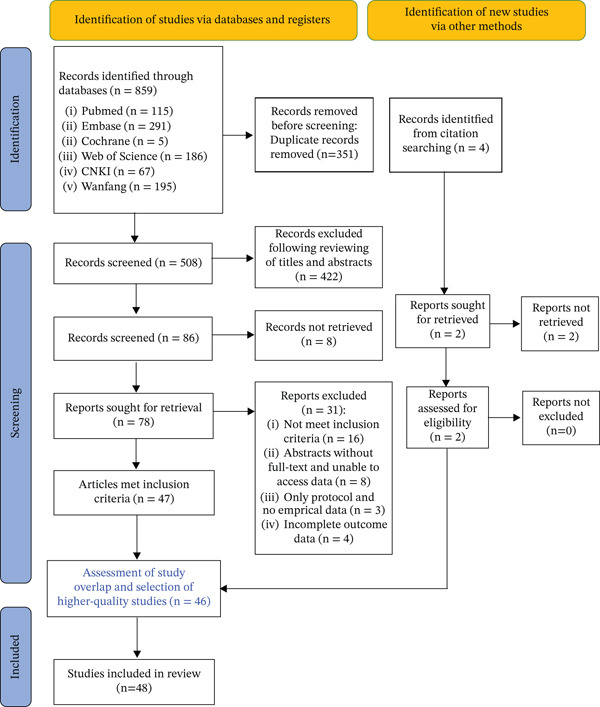
Flowchart of study selection process.

Our systematic search across six electronic databases (PubMed, Embase, Cochrane, Web of Science, CNKI, and Wanfang) initially identified 859 records. After removing 351 duplicates, 508 unique records were screened based on title and abstract. Following this initial screening, 422 irrelevant studies were excluded. The remaining 86 full‐text articles were assessed for eligibility, of which eight could not be retrieved. A total of 78 articles underwent full‐text review. Among these, 31 were excluded for the following reasons: not meeting inclusion criteria (*n* = 16), inaccessible full text or data (*n* = 8), protocol‐only publications without empirical data (*n* = 3), and incomplete outcome reporting (*n* = 4). Additionally, four records were identified through citation searching and subjected to full‐text screening. Ultimately, 48 studies met the inclusion criteria and were included in the systematic review (Figure 1).

### 3.2. Basic Characteristics of Included Studies

The included studies represent diverse study designs, geographical origins, and participant characteristics (Table S1**)**. Of the 48 studies, most were cross‐sectional studies [19–47], followed by cohort studies [48–62], and fewer case‐control studies [63–66]. Geographically, the largest contribution came from China, with additional studies from the United States, Japan, Australia, Europe, and other regions including Turkey, India, and Brazil. Sample sizes varied widely, ranging from under 100 to over 1 million participants [51]. Most studies involved older adults with Type 2 diabetes, with mean ages typically between 50–75 years. Commonly reported metrics included diabetes duration (median 5–15 years) and HbA1c (around 7%–9%).

DR was identified using ophthalmologic examinations, retinal photography, ICD codes, or ETDRS criteria. Cognitive function was evaluated with tools such as the MMSE, MoCA, and specialized tests including the TMT and DSST. Studies consistently adjusted for confounders including age, sex, education, diabetes duration, HbA1c, hypertension, cardiovascular disease, and other microvascular complications. Some also accounted for APOE *ε*4 genotype, depression, and socioeconomic factors.

### 3.3. Quality Assessment of Included Studies

The quality of the 29 cross‐sectional studies was evaluated using a standardized checklist (Table S2). Although most studies clearly defined their data sources and eligibility criteria, several failed to specify the patient identification period, consecutive enrollment, or evaluator masking. Descriptions of quality assurance and missing data handling were also limited. Overall, these studies demonstrated moderate methodological quality, with frequent shortcomings in blinding, confounder adjustment, and data transparency.

Case‐control (*n* = 4) and cohort (*n* = 15) studies were assessed using a star‐based system across selection, comparability, and exposure/outcome domains (Table S3). Most received high scores in selection (★★★–★★★★) and comparability (★★), with greater variation in exposure/outcome ratings (★–★★★). The majority were rated as high quality, bolstered by well‐defined selection criteria, adjustment for key confounders (e.g., age, diabetes duration, and HbA1c), and reliable outcome assessment. Overall, these longitudinal designs exhibited stronger methodological rigor compared with the cross‐sectional studies.

### 3.4. DR and Overall Cognitive Impairment

**Figure 2:**
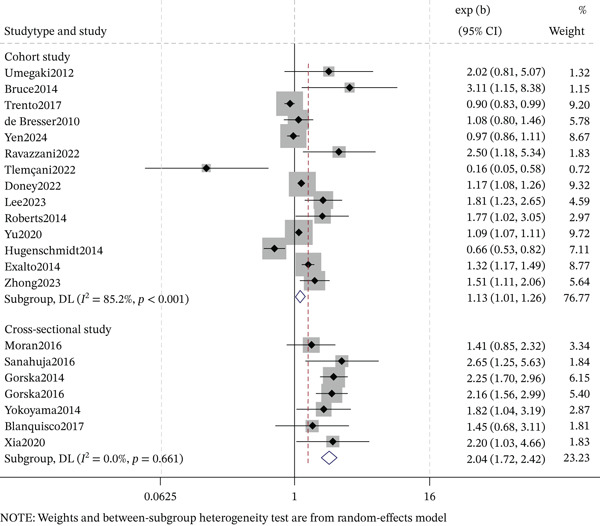
Forest plot and pooled estimates for the association between diabetic retinopathy and cognitive impairment.

The meta‐analysis examining the association between DR and cognitive impairment incorporated diverse studies and revealed significant heterogeneity overall **(**Figure 2). When stratified by study design, both the magnitude and direction of associations varied considerably. Among cross‐sectional studies, the pooled OR was 2.04 (95% CI: 1.72–2.42), with no evidence of heterogeneity (*I* ^2^ = 0.0*%*, *p* = 0.661). Among cohort studies, the pooled OR was 1.13 (95% CI: 1.01–1.26), with substantial heterogeneity (*I* ^2^ = 85.2*%*, *p* < 0.001).

### 3.5. DR and Cognitive Impairment Subtypes

**Figure 3:**
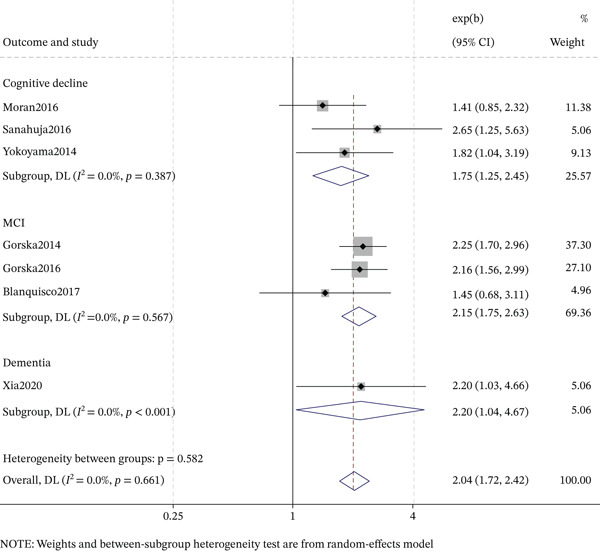
Forest plot and pooled estimates for cross‐sectional association between diabetic retinopathy and cognitive impairment subtypes.

**Figure 4:**
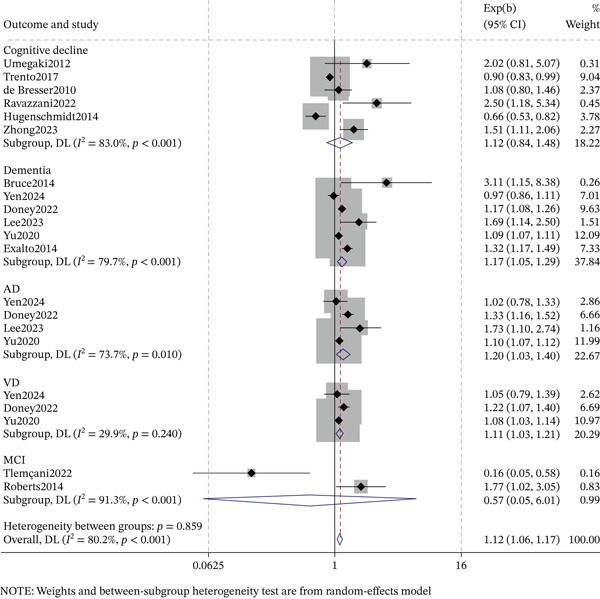
Forest plot and pooled estimates for longitudinal association between diabetic retinopathy and cognitive impairment subtypes.

The association between DR and cognitive impairment subtypes varied by study design (Figures 3 and 4). For cognitive decline, cross‐sectional studies showed a significant association (OR = 1.75, 95% CI: 1.25–2.45, *p* < 0.001; *I* ^2^ = 0.0*%*), but cohort studies did not (OR = 1.12, 95% CI: 0.84–1.48, *p* = 0.448; *I* ^2^ = 83.0*%*). For MCI, cross‐sectional studies demonstrated a strong association (OR = 2.15, 95% CI: 1.75–2.63, *p* < 0.001; *I* ^2^ = 0.0*%*), whereas cohort studies did not (OR = 0.57, 95% CI: 0.05–6.01, *p* = 0.642; *I* ^2^ = 91.3*%*). For dementia, significant associations were observed in both cross‐sectional (OR = 2.20, 95% CI: 1.04–4.67, *p* = 0.040; *I* ^2^ = 0.0*%*) and cohort studies (OR = 1.17, 95% CI: 1.05–1.29, *p* = 0.003; *I* ^2^ = 79.7*%*). In cohort studies, AD (OR = 1.20, 95% CI: 1.03–1.40, *I* ^2^ = 73.7*%*) and VD (OR = 1.11, 95% CI: 1.03–1.21, *I* ^2^ = 29.9*%*) were also significantly associated with DR (both *p* < 0.05). Overall, cross‐sectional studies supported associations with cognitive decline, MCI, and dementia, whereas longitudinal evidence supported associations with dementia, AD, and VD.

### 3.6. Subgroup Analysis on the Relationship Between DR and Cognitive Impairment

#### 3.6.1. Duration of Diabetes

**Figure 5:**
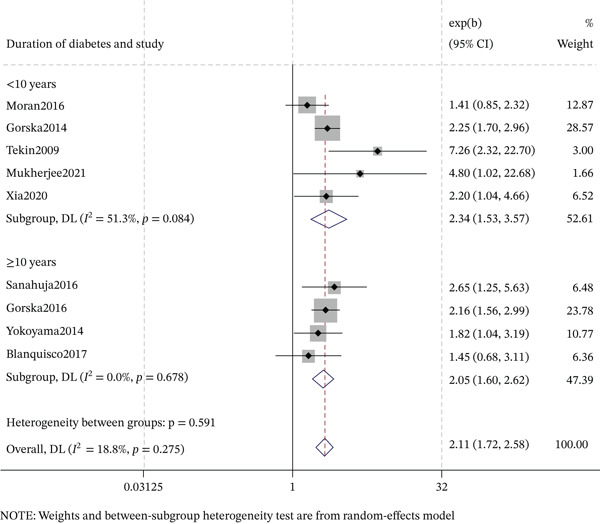
Forest plot of the subgroup analysis for cross‐sectional association between DR and cognitive impairment by duration of diabetes.

**Figure 6:**
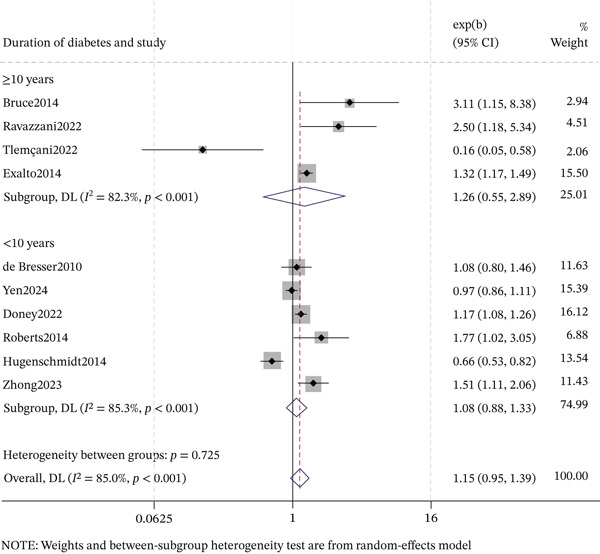
Forest plot of the subgroup analysis for longitudinal association between DR and cognitive impairment by duration of diabetes.

The association between DR and cognitive impairment stratified by diabetes duration (Figures 5 and 6). In cross‐sectional studies, both shorter (< 10 years) and longer (≥ 10 years) duration subgroups showed significant associations: < 10 years (OR = 2.34, 95% CI: 1.53–3.57, *p* < 0.001; *I* ^2^ = 51.3*%*) and ≥ 10 years (OR = 2.05, 95% CI: 1.60–2.62, *p* < 0.001; *I* ^2^ = 0.0*%*). In cohort studies, neither duration subgroup reached statistical significance: < 10 years (OR = 1.08, 95% CI: 0.88–1.33, *p* = 0.467; *I* ^2^ = 85.3*%*) and ≥ 10 years (OR = 1.26, 95% CI: 0.55–2.89, *p* = 0.588; *I* ^2^ = 82.3*%*). Collectively, the significant duration‐dependent association was driven by cross‐sectional studies, not by cohort studies.

#### 3.6.2. Follow‐Up Duration

**Figure 7:**
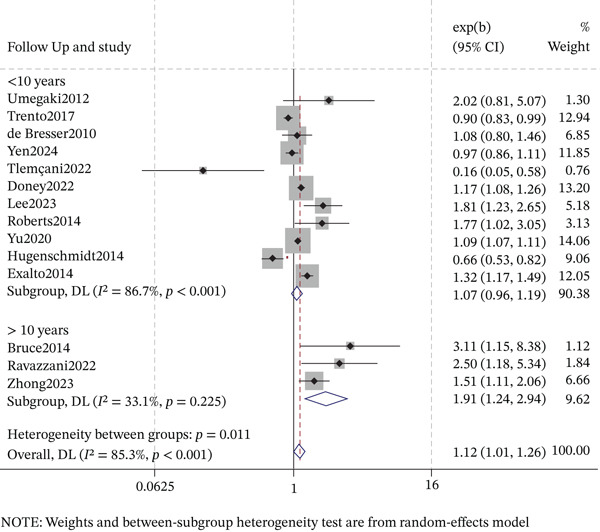
Forest plot of the subgroup analysis for the association between DR and cognitive impairment by follow‐up duration.

For studies with shorter follow‐up (< 10 years; *n* = 11), the pooled OR was not statistically significant (OR = 1.07, 95% CI: 0.96–1.19), with substantial heterogeneity (*I* ^2^ = 86.7*%*, *p* < 0.001) (Figure 7). Conversely, the subgroup with longer follow‐up (≥ 10 years; *n* = 3) showed a significantly stronger association (OR = 1.91, 95% CI: 1.24–2.94), with low heterogeneity (*I* ^2^ = 33.1*%*, *p* = 0.23). Collectively, these results indicate that the association between DR and cognitive impairment intensifies with extended observation periods, yielding a more pronounced effect estimate in studies with longer follow‐up.

#### 3.6.3. Cognitive Assessment Tools

**Figure 8:**
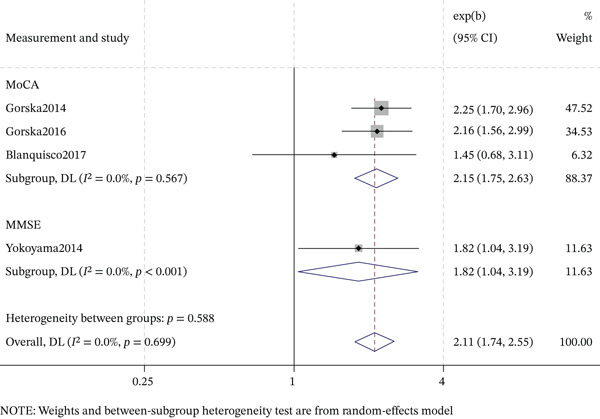
Forest plot of the subgroup analysis for cross‐sectional association between DR and cognitive impairment by cognitive assessment tool.

**Figure 9:**
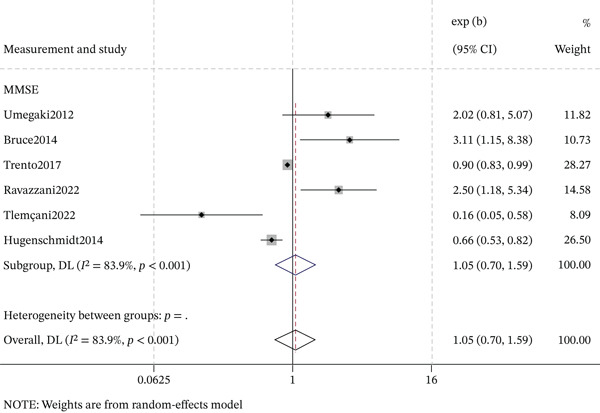
Forest plot of the subgroup analysis for longitudinal association between DR and cognitive impairment by cognitive assessment tools.

The association between DR and cognitive impairment varied by cognitive assessment tool (Figures 8 and 9). For MMSE, cross‐sectional studies showed a significant association (OR = 1.82, 95% CI: 1.04–3.19; *I* ^2^ = 0.0*%*), whereas cohort studies did not (OR = 1.05, 95% CI: 0.70–1.59; *I* ^2^ = 83.9*%*). For MoCA, cross‐sectional studies demonstrated a strong, consistent association (OR = 2.15, 95% CI: 1.75–2.63; *I* ^2^ = 0.0*%*). These results suggest that the estimated association is influenced by the choice of cognitive screening instrument and study design, with MoCA showing a robust positive effect in cross‐sectional studies.

### 3.7. Metaregression Analysis

To explore the sources of heterogeneity in cohort studies, we used a random‐effects metaregression model with logOR as the dependent variable, incorporating diabetes duration (≥ 10 years vs. < 10 years), follow‐up duration (≥ 10 years vs. < 10 years), region (Asia vs. Europe/America vs. others), and sample size (> 1000 vs. ≤ 1000) as covariates. The results showed that follow‐up duration significantly moderated the effect size (coefficient = −1.61, 95% CI: −2.66 to −0.57, *p* = 0.003), suggesting that studies with longer follow‐up had higher risk estimates compared with those with shorter follow‐up. None of the other covariates were statistically significant (all p > 0.05). However, the residual heterogeneity test remained significant (*Q* = 30.65, df = 4, *p* < 0.0001), suggesting that other unknown factors may also be influencing the results.

### 3.8. Sensitivity Analysis

To evaluate robustness, we performed leave‐one‐out analyses (fixed‐effect for cross‐sectional, random‐effects for cohort studies). For cross‐sectional studies, the pooled logOR was −0.34 (95% CI: −0.61 to −0.13); after omitting any single study, logOR ranged from −0.43 to −0.27, with all 95% CIs below zero (Figures S1 and S2). For cohort studies, the pooled logOR was 0.12 (95% CI: 0.01–0.23); leave‐one‐out estimates ranged from 0.09 to 0.16 (Figures S3 and S4). No single study altered the direction or magnitude of the association. Additionally, we compared DL with REML models (Tables S4–S8). For cross‐sectional studies, both models gave identical results. For cohort studies, DL yielded a significant association for overall cognitive impairment (OR = 1.13, *p* = 0.03), whereas REML was nonsignificant (OR = 1.19, *p* = 0.09). For cognitive subtypes and all subgroup analyses (diabetes duration, follow‐up, assessment tool), DL and REML estimates were consistent in direction and significance, with REML occasionally producing wider CIs but not altering conclusions. The REML sensitivity analysis confirms the robustness of the primary DL results.

### 3.9. Publication Bias

Publication bias was assessed only for cohort studies (*n* = 14), as the number of cross‐sectional studies was insufficient (< 10) for reliable tests. Visual inspection of the funnel plot and enhanced funnel plot revealed mild asymmetry, with a relative absence of small studies on the left side of the pooled effect, suggesting potential publication bias favoring positive results (Figures S5 and S6). Egger′s regression test indicated small‐study effects (slope coefficient = 0.08, 95% CI: 0.03–0.14, *p* = 0.01), whereas the bias intercept was not significant (coefficient = 0.37, 95% CI: –1.46 to 2.20, *p* = 0.67). Begg′s test also showed no evidence of publication bias (*p* = 0.70). To further adjust for potential bias, a nonparametric trim‐and‐fill analysis was performed, which imputed two hypothetically missing studies on the left side. The observed pooled logOR was 0.18 (95% CI: –0.03 to 0.38) under the random‐effects model, and after imputation it decreased to 0.11 (95% CI: –0.11 to 0.33) (Table S9). The overall conclusion remained unchanged, indicating that although some publication bias may be present, it does not substantially alter the association between DR and cognitive impairment in cohort studies.

## 4. Discussion

This comprehensive meta‐analysis of 48 studies confirms a significant association between DR and cognitive impairment in T2DM, with marked differences by study design. Cross‐sectional studies showed a two‐fold increased odds (OR = 2.04, 95% CI: 1.72–2.42; *I* ^2^ = 0.0*%*), whereas cohort studies showed a modest but significant association (OR = 1.13, 95% CI: 1.01–1.26; *I* ^2^ = 85.2*%*). This variability may be attributed to differences in diabetes duration, follow‐up duration, cognitive assessment tools, cognitive subtypes, and inherent design limitations (e.g., reverse causality in cross‐sectional studies, varying follow‐up in cohorts).

A key innovation is the detailed stratification of cognitive outcomes by study design. Cross‐sectionally, DR showed strong associations with MCI (OR = 2.15) and dementia (OR = 2.20), and a significant association with cognitive decline (OR = 1.75). Longitudinally, significant associations were only observed for dementia (OR = 1.17), AD (OR = 1.20), and VD (OR = 1.11), but not for cognitive decline or MCI. This refined analysis reveals that the DR‐dementia link is robust, whereas cross‐sectional findings for milder phenotypes may reflect reverse causality or detection bias. Thus, DR could serve as a research‐level risk signal for cognitive impairment, particularly dementia, in T2DM. However, given the observational nature and substantial heterogeneity in outcome definitions, measurement tools, and populations, prospective cohort studies with standardized protocols are urgently needed to validate these associations and determine their clinical relevance.

The association between DR and cognitive impairment may be explained through shared neurovascular injury mechanisms [67]. Embryologically and anatomically, the retina serves as an extension of the diencephalon, sharing structural and functional characteristics with the brain, including a specialized blood‐retinal barrier analogous to the blood‐brain barrier. Dysfunction of these barriers under conditions of chronic hyperglycemia and systemic inflammation facilitates the infiltration of cytokines, promoting cerebral insulin resistance and impairing neurovascular coupling. These processes disrupt synaptic plasticity, compromise neuronal survival, and hinder amyloid‐*β* clearance, ultimately leading to neurodegeneration [68, 69]. Retinal ganglion cells, which transmit visual information to the cortex, may both reflect and contribute to central nervous system degeneration [70]. Thus, retinal imaging offers a noninvasive window into cerebral microvascular health, potentially providing biomarkers for the early detection of cognitive decline in T2DM populations. Our results align with existing evidence indicating that retinal microvascular damage correlates with accelerated cognitive decline, reinforcing the role of DR as an indicator of generalized neurovascular pathology.

The temporal associations observed in this study further underscore the novelty of the findings, particularly when combined with the results on cognitive subtypes. This association was more pronounced among patients with a longer duration of diabetes (≥ 10 years) and in studies with longer follow‐up periods (≥ 10 years). Metaregression analysis confirmed that follow‐up duration is an independent moderator of this association (coefficient = –1.614, *p* = 0.003). Notably, longitudinal evidence supports significant associations only with dementia, AD, and VD, but shows no association with cognitive decline or MCI. In contrast, cross‐sectional studies showed strong associations with MCI and cognitive decline, suggesting that a dose‐response pattern—where long‐term exposure to diabetic microvascular damage increases cognitive risk—may be most pronounced in clinically defined dementia rather than in milder or preclinical phenotypes. This discrepancy suggests that findings from cross‐sectional studies targeting milder outcome measures may be influenced by reverse causality or detection bias, whereas the observed temporal relationships regarding dementia and its subtypes support the potential for chronic microvascular damage to have a causal role.

The temporal relationship reinforces the research‐level relevance of our findings, particularly when integrated with cognitive subtype results. The association was stronger with longer diabetes duration (≥ 10 years) and extended follow‐up (≥ 10 years), and meta‐regression confirmed follow‐up duration as an independent modifier (coefficient = –1.614, *p* = 0.003). Notably, longitudinal evidence supported significant associations only with dementia, AD, and VD, but not with milder phenotypes. In contrast, cross‐sectional studies showed strong associations with MCI and cognitive decline, suggesting that the dose‐response pattern may be most robust for overt dementia.

These results align with prior studies [71, 72] indicating that late‐onset diabetes confers lower cognitive risk, as complications develop over time. A key implication is that diabetes duration is a critical determinant of cognitive risk and should be incorporated into future studies, especially when dementia is the outcome. This is supported by a recent review identifying longer diabetes duration and DR as risk factors for progression from MCI to dementia [73], and by longitudinal evidence linking progressive DR to cognitive decline and dementia [74]. Microvascular retinal abnormalities are also linked to vascular cognitive impairment [75]. Given the stronger association with diabetes duration ≥ 10 years, future prospective studies should specifically examine whether DR status at this timepoint predicts incident dementia independently of other microvascular complications, and whether incorporating cognitive assessment into DR follow‐up protocols could enhance risk stratification in research settings.

Finally, the association between DR and cognitive impairment varied notably by the cognitive assessment tool used. MoCA‐based studies consistently demonstrated a strong positive association with negligible heterogeneity (OR = 2.11, 95% CI: 1.74–2.55, *I* ^2^ = 0.0*%*). In contrast, MMSE‐based studies yielded a modest, nonsignificant pooled estimate with substantial heterogeneity (OR = 1.05, 95% CI: 0.70–1.59, *I* ^2^ = 83.9*%*). Although this pattern may imply that DR is more closely linked to executive function and attention deficits—domains better captured by the MoCA—methodological differences across studies cannot be excluded. The high heterogeneity in the MMSE subgroup likely reflects variations in diagnostic thresholds, population characteristics, study settings, and measurement practices. The absence of heterogeneity in the MoCA subgroup, while striking, may be due to more uniform study protocols. Therefore, the apparent advantage of MoCA over MMSE in detecting DR‐related cognitive deficits should be interpreted cautiously, as it may be confounded by methodological heterogeneity rather than true differences in domain sensitivity. Head‐to‐head comparisons of MoCA and MMSE within the same prospective cohort, using standardized protocols, are needed to determine whether the observed differences reflect instrument‐specific psychometric properties (e.g., ceiling effects, domain coverage) or between‐study heterogeneity.

Several limitations of this meta‐analysis should be noted. First, despite extensive subgroup analyses, substantial unexplained heterogeneity (*I* ^2^ > 85*%*) persisted, reflecting variations in populations, diagnostic criteria, and study designs. Second, causal inference is limited by the predominance of cross‐sectional studies; future large‐scale, long‐term prospective cohorts with repeated retinal and cognitive assessments are needed. Third, residual confounding by unmeasured factors (e.g., socioeconomic status, genetics, and lifestyle) remains possible, and future studies should adopt propensity score matching or Mendelian randomization. Fourth, publication bias may exist, particularly among cohort studies: Egger′s test suggested small‐study effects. The trim‐and‐fill method imputed two missing studies, changing the pooled logOR from −0.175 to 0.111 (both 95% CIs included zero). However, this method assumes symmetry and may not fully correct bias; therefore, prospective registered cohorts are warranted to confirm the findings.

## 5. Conclusion

The meta‐analysis confirms a significant association between DR and cognitive impairment in T2DM, but the magnitude varies substantially by cognitive subtype and study design. Cross‐sectional studies show strong associations with MCI (OR = 2.15) and cognitive decline (OR = 1.75), whereas longitudinal evidence supports significant associations only with dementia (OR = 1.17), AD (OR = 1.20), and VD (OR = 1.11), but not with milder phenotypes. The association is strengthened by longer diabetes duration and extended follow‐up (≥ 10 years), with metaregression confirming follow‐up duration as an independent moderator. These findings support DR as a practical auxiliary indicator for risk stratification of dementia in T2DM, reflecting shared cerebral microvascular pathology. Future prospective cohort studies with standardized cognitive phenotyping are needed to establish causality and validate whether incorporating DR status into routine assessments can improve the identification of high‐risk individuals for targeted preventive interventions.

## Author Contributions

Ye He and Yining Zhu contributed equally to this work; they were jointly responsible for formulating the search strategy, screening the literature, and drafting the manuscript. Lixiao Yang performed literature screening and manuscript revision. Yuzhu Wang carried out data extraction and manuscript review. Chuyi Tan participated in literature screening, data extraction, and result discussion. Fangfang Ma and Ying Xia designed the study, polished and revised the manuscript, and served as joint guarantors, taking full responsibility for the overall content. Ye He and Yining Zhu should be considered joint first authors. Fangfang Ma and Ying Xia should be considered joint senior authors.

## Funding

No funding was received for this manuscript.

## Disclosure

The authors declare that no external funding was received for the conduct of this study, the preparation of the manuscript, or the decision to submit it for publication. All authors have approved the final version of the submitted manuscript and agree to be accountable for all aspects of the work.

## Conflicts of Interest

The authors declare no conflicts of interest.

## Supporting information


**Supporting Information** Additional supporting information can be found online in the Supporting Information section. The supplementary methods are presented as follows: Table S1 lists the characteristics of the included studies. Tables S2 and S3 present the quality assessments of cross‐sectional studies and of case‐control/cohort studies, respectively. Tables S4–S8 report sensitivity analyses comparing DL and REML random‐effects models for overall cognitive impairment, cognitive subtypes, duration of diabetes, follow‐up duration, and cognitive assessment tools. Table S9 shows the pooled effect size from the trim‐and‐fill method in cohort studies. Table S10 is the PRISMA checklist. Figures S1 and S2 display sensitivity analysis results (fixed model) and leave‐one‐out analysis for cross‐sectional studies. Figures S3 and S4 show the corresponding sensitivity analysis (random model) and leave‐one‐out analysis for cohort studies. Figures S5 and S6 present the funnel plot and enhanced funnel plot of the standard error of log odds ratio for the association between diabetic retinopathy and cognitive impairment.

## Data Availability

The data that support the findings of this study are available from the corresponding authors upon reasonable request.

## References

[1] International Diabetes Federation , Over 250 Million People Worldwide Unaware They Have Diabetes, 2025, According to New Research From the International Diabetes Federation (IDF), Accessed April 7, 2025, https://www.prnewswire.com/news-releases/over-250-million-people-worldwide-unaware-they-have-diabetes-according-to-new-research-from-the-international-diabetes-federation-idf-302419968.html. 10.1016/j.diabres.2025.112176 40220795

[2] International Diabetes Federation , IDF Global Clinical Practice Recommendations for Managing Type 2 Diabetes, 2025, Accessed April 7, 2025, https://idf.org/media/uploads/2025/04/IDF_Rec_2025.pdf.

[3] NCD Risk Factor Collaboration (NCD-Ris C) , Worldwide Trends in Diabetes Prevalence and Treatment From 1990 to 2022: a Pooled Analysis of 1108 Population-Representative Studies With 141 Million Participants, Lancet. 2024, no. 404, 2077–2093. 10.1016/S0140-6736(24)02317-1 PMC7616842 39549716

[4] Cheung N. , Mitchell P. , and Wong T. Y. , Diabetic Retinopathy, Lancet. (2010) 376, no. 9735, 124–136, 10.1016/S0140-6736(09)62124-3, 2-s2.0-77955013602. 20580421

[5] GBD 2019 Blindness and Vision Impairment Collaborators; Vision Loss Expert Group of the Global Burden of Disease Study , Causes of Blindness and Vision Impairment in 2020 and Trends over 30 Years, and Prevalence of Avoidable Blindness in Relation to VISION 2020: the Right to Sight: An Analysis for the Global Burden of Disease Study, The Lancet Global Health. (2021) 9, e144–e160, 10.1016/S2214-109X(20)30489-7. 33275949 PMC7820391

[6] Chatterjee S. , Peters S. A. , Woodward M. , Mejia Arango S. , Batty G. D. , Beckett N. , Beiser A. , Borenstein A. R. , Crane P. K. , Haan M. , Hassing L. B. , Hayden K. M. , Kiyohara Y. , Larson E. B. , Li C. Y. , Ninomiya T. , Ohara T. , Peters R. , Russ T. C. , Seshadri S. , Strand B. H. , Walker R. , Xu W. , and Huxley R. R. , Type 2 Diabetes as a Risk Factor for Dementia in Women Compared With Men: A Pooled Analysis of 2·3 Million People Comprising More Than 100,000 Cases of Dementia, Diabetes Care. (2016) 39, no. 2, 300–307, 10.2337/dc15-1588, 2-s2.0-84962128814, 26681727. 26681727 PMC4722942

[7] Livingston G. , Huntley J. , Sommerlad A. , Ames D. , Ballard C. , Banerjee S. , Brayne C. , Burns A. , Cohen-Mansfield J. , Cooper C. , Costafreda S. G. , Dias A. , Fox N. , Gitlin L. N. , Howard R. , Kales H. C. , Kivimäki M. , Larson E. B. , Ogunniyi A. , Orgeta V. , Ritchie K. , Rockwood K. , Sampson E. L. , Samus Q. , Schneider L. S. , Selbæk G. , Teri L. , and Mukadam N. , Dementia Prevention, Intervention, and Care: 2020 report of the Lancet Commission, Lancet. (2020) 396, no. 10248, 413–446, 10.1016/S0140-6736(20)30367-6, 32738937. 32738937 PMC7392084

[8] Sun L. , Diao X. , Gang X. , Lv Y. , Zhao X. , Yang S. , Gao Y. , and Wang G. , Risk Factors for Cognitive Impairment in Patients With Type 2 Diabetes, Journal of Diabetes Research. (2020) 2020, 4591938, 10.1155/2020/4591938. 32377520 PMC7196145

[9] Marseglia A. , Fratiglioni L. , Kalpouzos G. , Wang R. , Bäckman L. , and Xu W. , Prediabetes and Diabetes Accelerate Cognitive Decline and Predict Microvascular Lesions: A Population-Based Cohort Study, Alzheimer′s & Dementia. (2019) 15, 25–33, 10.1016/j.jalz.2018.06.3060, 2-s2.0-85055417373. 30114414

[10] Roberts R. O. , Geda Y. E. , Knopman D. S. , Christianson T. J. , Pankratz V. S. , Boeve B. F. , Vella A. , Rocca W. A. , and Petersen R. C. , Association of Duration and Severity of Diabetes Mellitus With Mild Cognitive Impairment, Archives of Neurology. (2008) 65, no. 8, 1066–1073, 10.1001/archneur.65.8.1066, 2-s2.0-49449089294, 18695056. 18695056 PMC2630223

[11] Gupta P. , Gan A. T. L. , Man R. E. K. , Fenwick E. K. , Sabanayagam C. , Mitchell P. , Cheung C. Y. , Cheung N. , Wong T. Y. , Cheng C. Y. , and Lamoureux E. L. , Association Between Diabetic Retinopathy and Incident Cognitive Impairment, British Journal of Ophthalmology. (2019) 103, no. 11, 1605–1609, 10.1136/bjophthalmol-2018-312807, 2-s2.0-85060888327. 31645330

[12] Simó R. , Stitt A. W. , and Gardner T. W. , Neurodegeneration in Diabetic Retinopathy: Does It Really Matter?, Diabetologia. (2018) 61, no. 9, 1902–1912, 10.1007/s00125-018-4692-1, 2-s2.0-85050297507, 30030554. 30030554 PMC6096638

[13] Starr J. M. , Wardlaw J. , Ferguson K. , MacLullich A. , Deary I. J. , and Marshall I. , Increased Blood-Brain Barrier Permeability in Type II Diabetes Demonstrated by Gadolinium Magnetic Resonance Imaging, Journal of Neurology, Neurosurgery & Psychiatry. (2003) 74, 70–76. 12486269 10.1136/jnnp.74.1.70 PMC1738177

[14] Palacios A. G. , Zhang S. X. , and Acosta M. L. , Diabetic Retinopathy and Alzheimer′s Disease: Convergence of the Unfolded Protein Response in Neurodegeneration, Alzheimer′s & Dementia. (2025) 21, no. 8, e70497, 10.1002/alz.70497, 40760071. PMC12321509 40760071

[15] Crosby-Nwaobi R. R. , Sivaprasad S. , Amiel S. , and Forbes A. , The Relationship Between Diabetic Retinopathy and Cognitive Impairment, Diabetes Care. (2013) 36, no. 10, 3177–3186, 10.2337/dc12-2141, 2-s2.0-84891876985, 23633523. 23633523 PMC3781499

[16] Page M. J. , McKenzie J. E. , Bossuyt P. M. , Boutron I. , Hoffmann T. C. , Mulrow C. D. , Shamseer L. , Tetzlaff J. M. , Akl E. A. , Brennan S. E. , Chou R. , Glanville J. , Grimshaw J. M. , Hróbjartsson A. , Lalu M. M. , Li T. , Loder E. W. , Mayo-Wilson E. , McDonald S. , McGuinness L. A. , Stewart L. A. , Thomas J. , Tricco A. C. , Welch V. A. , Whiting P. , and Moher D. , The PRISMA 2020 Statement: An Updated Guideline for Reporting Systematic Reviews, Journal of Clinical Epidemiology. (2021) 134, 178–189, 10.1016/j.jclinepi.2021.03.001, 33789819. 33789819

[17] Stang A. , Critical Evaluation of the Newcastle-Ottawa scale for the Assessment of the Quality of Nonrandomized Studies in Meta-Analyses, European Journal of Epidemiology. (2010) 25, no. 9, 603–605, 10.1007/s10654-010-9491-z, 2-s2.0-77957661914, 20652370. 20652370

[18] Grant R. L. , Converting an Odds Ratio to a Range of Plausible Relative Risks for Better Communication of Research findings, BMJ. (2014) 348, f7450, 10.1136/bmj.f7450, 2-s2.0-84893097567, 24464277. 24464277

[19] Liu X. , Resting-State Functional Brain Magnetic Resonance Imaging Study in Type 2 Diabetic Retinopathy, 2016, Bengbu Medical University.

[20] Su Y. , Quantitative Evaluation of Brain White Matter Injury in Type 2 Diabetes Mellitus, 2021, Xi′an Medical University.

[21] Wu C. , Correlation Study of Cognitive Function Level and Diabetic Retinopathy and TCM Syndrome in Type 2 Diabetes Mellitus Patients, 2022, Gansu University Of Chinese Medicine.

[22] Xiao Y. , Analysis of the Relationship Between Diabetic Retinopathy and Cognitive Function in Type 2 Diabetes Mellitus, 2023, Hubei University of Medicine.

[23] Yang M. , Exploration of the Relationship Between Type 2 Diabetic Retinopathy and Mild Cognitive Impairment, 2023, Yan′an University.

[24] Zhu X. , Ji M. , Dai Z. , Chen F. , Pan X. , Pan Y. , Sun K. , and Jiang D. , A Study on the Relationship Between Retinopathy and Cognitive Function in Newly Diagnosed Type 2 Diabetes Patients, Medical Journal of Communications. (2019) 33, 15–18.

[25] Liu S. , Wang X. , Wang P. , Lixia H. , Longchun X. , and Lei Z. , Amplitude of Low Frequency Fluctuation in Resting-State Functional Magnetic Resonance Imaging for Patients With Type 2 Diabetes Retinopathy, Chinese Journal of Medical Physics. (2016) 33, 1085–1090.

[26] Finger R. P. , Fenwick E. , Cheung C. Y. , Ikram M. K. , Wong T. Y. , and Lamoureux E. L. , Near Vision Impairment Is Associated With Cognitive Impairment in Type 2 Diabetes, Asia-Pacific Journal of Ophthalmology. (2014) 3, no. 1, 17–22, 10.1097/APO.0b013e3182a4d1d5, 26107302. 26107302

[27] Moran C. , Tapp R. J. , Hughes A. D. , Magnussen C. G. , Blizzard L. , Phan T. G. , Beare R. , Witt N. , Venn A. , Münch G. , Amaratunge B. C. , and Srikanth V. , The Association of Type 2 Diabetes Mellitus With Cerebral Gray Matter Volume Is Independent of Retinal Vascular Architecture and Retinopathy, Journal of Diabetes Research. (2016) 2016, 9, 10.1155/2016/6328953, 2-s2.0-84975127162. PMC4897713 27314049

[28] Sanahuja J. , Alonso N. , Diez J. , Ortega E. , Rubinat E. , Traveset A. , Alcubierre N. , Betriu À. , Castelblanco E. , Hernández M. , Purroy F. , Arcidiacono M. V. , Jurjo C. , Fernández E. , Puig-Domingo M. , Groop P. H. , and Mauricio D. , Increased Burden of Cerebral Small Vessel Disease in Patients With Type 2 Diabetes and Retinopathy, Diabetes Care. (2016) 39, no. 9, 1614–1620, 10.2337/dc15-2671, 2-s2.0-84986182398, 27281772. 27281772

[29] Gorska-Ciebiada M. , Saryusz-Wolska M. , Ciebiada M. , and Loba J. , Mild cognitive Impairment and Depressive Symptoms in Elderly Patients With Diabetes: Prevalence, Risk Factors, and Comorbidity, Journal of Diabetes Research. (2014) 2014, 179648, 10.1155/2014/179648, 2-s2.0-84912550437. 25431771 PMC4241577

[30] Gorska-Ciebiada M. , Saryusz-Wolska M. , Borkowska A. , Ciebiada M. , and Loba J. , Adiponectin, Leptin and IL-1 *β* in Elderly Diabetic Patients With Mild Cognitive Impairment, Metabolic Brain Disease. (2016) 31, no. 2, 257–266, 10.1007/s11011-015-9739-0, 2-s2.0-84961121743, 26432692. 26432692 PMC4791470

[31] Yu Z. W. , Liu R. , Li X. , Wang Y. , Fu Y. H. , Li H. Y. , Yuan Y. , and Gao X. Y. , High Serum Neuron-Specific Enolase Level Is Associated With Mild Cognitive Impairment in Patients With Diabetic Retinopathy, Metabolic Syndrome and Obesity. (2020) 13, 1359–1365, 10.2147/DMSO.S249126, 32425568. PMC7188072 32425568

[32] Sajeev P. G. , Krishnagopal S. , and Subramanian K. , The Association Between Diabetic Retinopathy, Cognitive Impairment, and Quality of life—A Cross Sectional Study, Diabetes Epidemiology and Management. (2023) 11, 100142, 10.1016/j.deman.2023.100142.

[33] Umegaki H. , Iimuro S. , Shinozaki T. , Araki A. , Sakurai T. , Iijima K. , Ohashi Y. , and Ito H. , Risk Factors Associated With Abnormal Cognition in Japanese Outpatients With Diabetes, Hypertension or Dyslipidemia, Diabetology International. (2015) 6, no. 4, 268–274, 10.1007/s13340-014-0194-7, 2-s2.0-84949236586.

[34] Tong J. , Geng H. , Zhang Z. , Zhu X. , Meng Q. , Sun X. , Zhang M. , Qian R. , Sun L. , and Liang Q. , Brain Metabolite Alterations Demonstrated by Proton Magnetic Resonance Spectroscopy in Diabetic Patients With Retinopathy, Magnetic Resonance Imaging. (2014) 32, 1037–1042. 24985566 10.1016/j.mri.2014.04.020

[35] Gao Y. , Xiao Y. , Miao R. , Zhao J. , Cui M. , Huang G. , and Fei M. , The Prevalence of Mild Cognitive Impairment With Type 2 Diabetes Mellitus Among Elderly People in China: A Cross-Sectional Study, Archives of Gerontology and Geriatrics. (2016) 62, 138–142, 10.1016/j.archger.2015.09.003, 2-s2.0-84952987053, 26381432. 26381432

[36] Wang Z. L. , Zou L. , Lu Z. W. , Xie X. Q. , Jia Z. Z. , Pan C. J. , Zhang G. X. , and Ge X. M. , Abnormal Spontaneous Brain Activity in Type 2 Diabetic Retinopathy Revealed by Amplitude of Low-Frequency Fluctuations: A Resting-State fMRI Study, Clinical Radiology. (2017) 72, no. 4, 340.e1–340.e7, 10.1016/j.crad.2016.11.012, 2-s2.0-85009887596. 28041652

[37] Mukherjee S. , Ghosh S. , and Ghosh S. , Association of Midlife Cognition Impairment With Diabetic Retinopathy in Type 2 Diabetes Mellitus in an Indian Population, Practical Diabetes. (2022) 39, no. 2, 24–29, 10.1002/pdi.2385.

[38] Zhu X. , Jiang D. , Zhang H. , Cai R. , Wang Y. , and Hua F. , An Investigation of the Correlation Between Retinal Nerve Fiber Layer Thickness With Blood Biochemical Indices and Cognitive Dysfunction in Patients With Type 2 Diabetes Mellitus, Diabetes, Metabolic Syndrome and Obesity. (2024) 17, 3315–3323, 10.2147/DMSO.S470297, 39247429. PMC11380875 39247429

[39] Zheng M. , Zhang M. , Yang J. , Zhao S. , Qin S. , Chen H. , Gao Y. , and Huang G. , Relationship Between Blood Levels of Methyl Donor and Folate and Mild Cognitive Impairment in Chinese Patients With Type 2 Diabetes: A Case-Control Study, Journal of Clinical Biochemistry and Nutrition. (2014) 54, 122–128, 10.3164/jcbn.13-89, 2-s2.0-84897564607. 24688222 PMC3947971

[40] Roy S. , Kim N. , Desai A. , Komaragiri M. , Jassil N. , Khan M. , Cole R. , Desai N. , Terrigno R. , Hunter K. , Baxi N. , and Blessinger M. , Cognitive Function and Control of Type 2 Diabetes Mellitus in Young Adults, North American Journal of Medicine and Science. (2015) 7, no. 5, 220–226, 10.4103/1947-2714.157627, 2-s2.0-84982756959, 26110134. PMC4462818 26110134

[41] Ogama N. , Sakurai T. , Kawashima S. , Tanikawa T. , Tokuda H. , Satake S. , Miura H. , Shimizu A. , Kokubo M. , Niida S. , Toba K. , Umegaki H. , and Kuzuya M. , Association of Glucose Fluctuations With Sarcopenia in Older Adults With Type 2 Diabetes Mellitus, Journal of Clinical Medicine. (2019) 8, no. 3, 10.3390/jcm8030319, 30845785. PMC6463152 30845785

[42] Murata Y. , Kadoya Y. , Yamada S. , and Sanke T. , Cognitive Impairment in Elderly Patients With Type 2 diabetes Mellitus: Prevalence and Related Clinical Factors, Diabetology International. (2017) 8, no. 2, 193–198, 10.1007/s13340-016-0292-9, 2-s2.0-85019723275. 30603321 PMC6224960

[43] Blanquisco L. , Abejero J. E. , Buno B. , Trajano-Acampado L. , Cenina A. , and Santiago D. , Factors Associated With Mild Cognitive Impairment Among Elderly Filipinos With Type 2 Diabetes Mellitus, Journal of the ASEAN Federation of Endocrine Societies. (2017) 32, no. 2, 145–150, 10.15605/jafes.032.02.08, 2-s2.0-85035803645, 33442098. 33442098 PMC7784175

[44] Xia S. S. , Xia W. L. , Huang J. J. , Zou H. J. , Tao J. , and Yang Y. , The Factors Contributing to Cognitive Dysfunction in Type 2 Diabetic Patients, Annals of Translational Medicine. (2020) 8, no. 4, 10.21037/atm.2019.12.113, 32175397. PMC7049020 32175397

[45] Ding J. , Strachan M. W. , Reynolds R. M. , Frier B. M. , Deary I. J. , Fowkes F. G. , Lee A. J. , McKnight J. , Halpin P. , Swa K. , and Price J. F. , Diabetic Retinopathy and Cognitive Decline in Older People With Type 2 Diabetes: the Edinburgh Type 2 Diabetes Study, Diabetes. (2010) 59, no. 11, 2883–2889, 10.2337/db10-0752, 2-s2.0-78049299161, 20798334. 20798334 PMC2963547

[46] Kwa V. I. , van der Sande J. J. , Stam J. , Tijmes N. , Vrooland J. L. , and Amsterdam Vascular Medicine Group , Retinal Arterial Changes Correlate With Cerebral Small-Vessel Disease, Neurology. (2002) 59, no. 10, 1536–1540, 10.1212/01.WNL.0000033093.16450.5C, 2-s2.0-0037180475, 12451193. 12451193

[47] Erdener Ş. E. and Dalkara T. , Small Vessels Are a Big Problem in Neurodegeneration and Neuroprotection, Frontiers in Neurology. (2019) 10, 10.3389/fneur.2019.00889, 2-s2.0-85071735457, 31474933. PMC6707104 31474933

[48] Umegaki H. , Iimuro S. , Shinozaki T. , Araki A. , Sakurai T. , Iijima K. , Ohashi Y. , Ito H. , and the Japanese Elderly Diabetes Intervention Trial Study Group , Risk Factors Associated With Cognitive Decline in the Elderly With Type 2 Diabetes: Baseline Data Analysis of the Japanese Elderly Diabetes Intervention Trial, Geriatrics & Gerontology International. (2012) 12, no. s1, 103–109, 10.1111/j.1447-0594.2011.00817.x, 2-s2.0-84858782892, 22435945. 22435945

[49] Bruce D. G. , Davis W. A. , Starkstein S. E. , and Davis T. M. , Mid-life Predictors of Cognitive Impairment and Dementia in Type 2 Diabetes Mellitus: the Fremantle Diabetes Study, Journal of Alzheimer′s Disease. (2014) 42, no. s3, S63–S70, 10.3233/JAD-132654, 2-s2.0-84920626821, 24840567. 24840567

[50] Trento M. , Charrier L. , Salassa M. , Merlo S. , Passera P. , Baltatescu A. , Cavallo F. , and Porta M. , Cognitive Function May be a Predictor of Retinopathy Progression in Patients With Type 2 Diabetes, European Journal of Ophthalmology. (2017) 27, no. 3, 278–280, 10.5301/ejo.5000885, 2-s2.0-85019387547, 27716894. 27716894

[51] de Bresser J. , Reijmer Y. D. , van den Berg E. , Breedijk M. A. , Kappelle L. J. , Viergever M. A. , Biessels G. J. , and on behalf of the Utrecht Diabetic Encephalopathy Study Group , Microvascular Determinants of Cognitive Decline and Brain Volume Change in Elderly Patients With Type 2 DIABETES, Dementia and Geriatric Cognitive Disorders. (2010) 30, no. 5, 381–386, 10.1159/000321354, 2-s2.0-77958021269, 20962529. 20962529

[52] Yen Y. H. , Yen F. S. , Ko F. S. , Wei J. C. C. , Huang Y. , Yu T. S. , Hwu C. M. , and Hsu C. C. , Microvascular Disease and Its Association With Dementia in Patients With Type 2 Diabetes: A Nationwide Cohort Study in Taiwan, Diabetes, Obesity and Metabolism. (2024) 26, no. 11, 5399–5407, 10.1111/dom.15908, 39210562. 39210562

[53] de Almeida Faria A. C. R. , Dall′Agnol J. F. , Gouveia A. M. , de Paiva C. I. , Segalla V. C. , and Baena C. P. , Risk Factors for Cognitive Decline in Type 2 Diabetes Mellitus Patients in Brazil: A Prospective Observational Study, Diabetology & Metabolic Syndrome. (2022) 14, no. 1, 10.1186/s13098-022-00872-3, 35897033. PMC9327152 35897033

[54] Rhmari Tlemçani F. Z. , Elamari S. , Motaib I. , Laidi S. , Alidrissi N. , Ahid S. , and Chadli A. , Factors Associated With Mild Cognitive Impairment in Patients With Type 2 Diabetes: A Cohort Study, Cureus. (2022) 14, e28305, 10.7759/cureus.28305. 36168366 PMC9506426

[55] Doney A. S. F. , Nar A. , Huang Y. , Trucco E. , MacGillivray T. , Connelly P. , Leese G. P. , McKay G. J. , and on behalf of the INSPIRED consortium , Retinal Vascular Measures From Diabetes Retinal Screening Photographs and Risk of Incident Dementia in Type 2 Diabetes: A GoDARTS Study, Frontiers in Digital Health. (2022) 4, 945276, 10.3389/fdgth.2022.945276, 36120710. 36120710 PMC9470757

[56] Lee C. S. , Krakauer C. , Su Y. R. , Walker R. L. , Blazes M. , McCurry S. M. , Bowen J. D. , McCormick W. C. , Lee A. Y. , Boyko E. J. , O′Hare A. M. , Larson E. B. , and Crane P. K. , Diabetic Retinopathy and Dementia Association, Beyond Diabetes Severity, Beyond Diabetes Severity, American Journal of Ophthalmology. (2023) 249, 90–98, 10.1016/j.ajo.2022.12.003, 36513155. 36513155 PMC10106379

[57] Roberts R. O. , Knopman D. S. , Geda Y. E. , Cha R. H. , Pankratz V. S. , Baertlein L. , Boeve B. F. , Tangalos E. G. , Ivnik R. J. , Mielke M. M. , and Petersen R. C. , Association of Diabetes With Amnestic and Nonamnestic Mild Cognitive Impairment, Alzheimer′s & Dementia. (2014) 10, 18–26. 10.1016/j.jalz.2013.01.001 PMC3830601 23562428

[58] Hendrie H. C. , Zheng M. , Lane K. A. , Ambuehl R. , Purnell C. , Li S. , Unverzagt F. W. , Murray M. D. , Balasubramanyam A. , Callahan C. M. , and Gao S. , Changes of Glucose Levels Precede Dementia in African-Americans With Diabetes But Not in Caucasians, Alzheimer′s & Dementia. (2018) 14, no. 12, 1572–1579, 10.1016/j.jalz.2018.03.008, 2-s2.0-85057416694, 29678640. PMC6192866 29678640

[59] Yu J. H. , Han K. , Park S. , Cho H. , Lee D. Y. , Kim J. W. , Seo J. A. , Kim S. G. , Baik S. H. , Park Y. G. , Choi K. M. , Kim S. M. , and Kim N. H. , Incidence and Risk Factors for Dementia in Type 2 Diabetes Mellitus: A Nationwide Population-Based Study in Korea, Diabetes & Metabolism Journal. (2020) 44, 113–124. 31769236 10.4093/dmj.2018.0216 PMC7043975

[60] Hugenschmidt C. E. , Lovato J. F. , Ambrosius W. T. , Bryan R. N. , Gerstein H. C. , Horowitz K. R. , Launer L. J. , Lazar R. M. , Murray A. M. , Chew E. Y. , Danis R. P. , Williamson J. D. , Miller M. E. , and Ding J. , The Cross-Sectional and Longitudinal Associations of Diabetic Retinopathy With Cognitive Function and Brain MRI Findings: The Action to Control Cardiovascular Risk in Diabetes (ACCORD) Trial, Diabetes Care. (2014) 37, no. 12, 3244–3252, 10.2337/dc14-0502, 2-s2.0-84911874575, 25193529. 25193529 PMC4237980

[61] Exalto L. G. , Biessels G. J. , Karter A. J. , Huang E. S. , Quesenberry C. P. , and Whitmer R. A. , Severe Diabetic Retinal Disease and Dementia Risk in Type 2 Diabetes, Journal of Alzheimer′s Disease. (2014) 42, no. s3, S109–S117, 10.3233/JAD-132570, 2-s2.0-84912100524, 24625797. PMC4373321 24625797

[62] Zhong P. , Tan S. , Zhu Z. , Zhang J. , Chen S. , Huang W. , He M. , and Wang W. , Brain and Cognition Signature Fingerprinting Vascular Health in Diabetic Individuals: An International Multi-Cohort Study, American Journal of Geriatric Psychiatry. (2023) 31, no. 8, 570–582, 10.1016/j.jagp.2023.04.010, 37230837. 37230837

[63] Tekn O. , Ukur S. , Karadag R. , Tunca A. , Göktaş O. , Özkara A. , Işik B. , Cebeci S. , and Yiğitoğlu R. , Cognitive Impairment Among Type-2 Diabetic Subjects and Its Relationship With Long-Term Complications, Turkish Journal of Medical Sciences. (2009) 39, 703–710, 10.3906/sag-0803-18, 2-s2.0-70350432976.

[64] Naidu V. V. , Ismail K. , Amiel S. , Kohli R. , Crosby-Nwaobi R. , Sivaprasad S. , and Stewart R. , Associations Between Retinal Markers of Microvascular Disease and Cognitive Impairment in Newly Diagnosed Type 2 Diabetes Mellitus: A Case Control Study, PLoS One. (2016) 11, no. 1, e0147160, 10.1371/journal.pone.0147160, 2-s2.0-84955299837, 26771382. 26771382 PMC4714814

[65] Lu X. , Gong W. , Wen Z. , Hu L. , Peng Z. , and Zha Y. , Correlation Between Diabetic Cognitive Impairment and Diabetic Retinopathy in Patients With T2DM by 1H-MRS, Frontiers in Neurology. (2019) 10, 10.3389/fneur.2019.01068, 31781013. PMC6861416 31781013

[66] Maimaitituerxun R. , Chen W. , Xiang J. , Xie Y. , Xiao F. , Wu X. Y. , Chen L. , Yang J. , Liu A. , and Dai W. , Predictive Model for Identifying Mild Cognitive Impairment in Patients With Type 2 Diabetes Mellitus: A CHAID Decision Tree Analysis, Brain and Behavior. (2024) 14, no. 3, e3456, 10.1002/brb3.3456, 38450963. 38450963 PMC10918605

[67] Srikanth V. , Sinclair A. J. , Hill-Briggs F. , Moran C. , and Biessels G. J. , Type 2 Diabetes and Cognitive Dysfunction—Towards Effective Management of Both Comorbidities, Lancet Diabetes & Endocrinology. (2020) 8, no. 6, 535–545, 10.1016/S2213-8587(20)30118-2, 32445740. 32445740

[68] Biessels G. J. and Despa F. , Cognitive Decline and Dementia in Diabetes Mellitus: Mechanisms and Clinical Implications, Nature Reviews Endocrinology. (2018) 14, no. 10, 591–604, 10.1038/s41574-018-0048-7, 2-s2.0-85050161551, 30022099. PMC6397437 30022099

[69] Blazes M. and Lee C. S. , Understanding the Brain Through Aging Eyes, Advances in Geriatric Medicine and Research. (2021) 3, e210008. 33748826 10.20900/agmr20210008 PMC7971450

[70] Xu W. , Qiu C. , Gatz M. , Pedersen N. L. , Johansson B. , and Fratiglioni L. , Mid- and Late-Life Diabetes in Relation to the Risk of Dementia: A population-Based Twin Study, Diabetes. (2009) 58, no. 1, 71–77, 10.2337/db08-0586, 2-s2.0-63249111096, 18952836. 18952836 PMC2606895

[71] Pal K. , Mukadam N. , Petersen I. , and Cooper C. , Mild Cognitive Impairment and Progression to Dementia in People With Diabetes, Prediabetes and Metabolic Syndrome: A Systematic Review and Meta-Analysis, Social Psychiatry and Psychiatric Epidemiology. (2018) 53, no. 11, 1149–1160, 10.1007/s00127-018-1581-3, 2-s2.0-85053411808, 30182156. 30182156 PMC6208946

[72] Zilkens R. R. , Davis W. A. , Spilsbury K. , Semmens J. B. , and Bruce D. G. , Earlier Age of Dementia Onset and Shorter Survival Times in Dementia Patients With Diabetes, American Journal of Epidemiology. (2013) 177, no. 11, 1246–1254, 10.1093/aje/kws387, 2-s2.0-84878541187, 23543134. 23543134

[73] Hallaj S. , Halfpenny W. , Chuter B. G. , Weinreb R. N. , Baxter S. L. , and Cui Q. N. , Association Between Glucagon-Like Peptide-1 Receptor Agonists Exposure and Intraocular Pressure Change: GLP-1 Receptor Agonists and Intraocular Pressure Change, American Journal of Ophthalmology. (2025) 269, 255–265, 10.1016/j.ajo.2024.08.030, 39237049. 39237049 PMC11634659

[74] Tan L. , Wang Z. , Okoth K. , Toulis K. A. , Denniston A. K. , Singh B. M. , Crowe F. L. , Sainsbury C. , Wang J. , and Nirantharakumar K. , Associations of Antidiabetic Drugs With Diabetic Retinopathy in People With Type 2 Diabetes: an Umbrella Review and Meta-Analysis, Frontiers in Endocrinology. (2024) 14, 1303238, 10.3389/fendo.2023.1303238, 38239984. 38239984 PMC10795175

[75] Eleftheriadou A. , Riley D. , Zhao S. S. , Austin P. , Hernández G. , Lip G. Y. H. , Jackson T. L. , Wilding J. P. H. , and Alam U. , Risk of Diabetic Retinopathy and Diabetic Macular Oedema With Sodium-Glucose Cotransporter 2 Inhibitors and Glucagon-Like Peptide 1 Receptor Agonists in Type 2 Diabetes: A Real-World Data Study From a Global Federated Database, Diabetologia. (2024) 67, no. 7, 1271–1282, 10.1007/s00125-024-06132-5, 38584180. 38584180 PMC11153282

