# Supplementary material for: Diabetic Retinopathy and Cognitive Dysfunction in Type 2 Diabetes Mellitus: A Systematic Review and Meta‐Analysis of Epidemiological Associations and Clinical Implications
Source: J Diabetes Res. 2026 May 17;2026:1328324. doi: 10.1155/jdr/1328324 (PMC13181155; doi:10.1155/jdr/1328324)
Supplement: Supplementary file 1 — Supporting Information Additional supporting information can be found online in the Supporting Information section. The supplementary methods are presented as follows: Table S1 lists the characteristics of the included studies. Tables S2 and S3 present the quality assessments of cross‐sectional studies and of case‐control/cohort studies, respectively. Tables S4–S8 report sensitivity analyses comparing DL and REML random‐effects models for overall cognitive impairment, cognitive subtypes, duration of diabetes, follow‐up duration, and cognitive assessment tools. Table S9 shows the pooled effect size from the trim‐and‐fill method in cohort studies. Table S10 is the PRISMA checklist. Figures S1 and S2 display sensitivity analysis results (fixed model) and leave‐one‐out analysis for cross‐sectional studies. Figures S3 and S4 show the corresponding sensitivity analysis (random model) and leave‐one‐out analysis for cohort studies. Figures S5 and S6 present the funnel plot and enhanced funnel plot of the standard error of log odds ratio for the association between diabetic retinopathy and cognitive impairment. [file JDR-2026-1328324-s001.docx]

**Supplementary Material**

**Title: Diabetic Retinopathy and Cognitive Dysfunction in Type 2 Diabetes Mellitus: A Systematic Review and Meta-Analysis of Epidemiological Associations and Clinical Implications**

**Supplementary methods..........................................................................................................1**

**Supplement Table legends**

**Table S1. Characteristics of the studies included in the review...........................................3**

**Table S2. The quality of cross-sectional studies..................................................................13**

**Table S3. The quality of case-control studies or cohort studies.........................................16**

**Table S4. Sensitivity analysis – comparison of DL vs. REML models for meta-analysis of overall cognitive impairment by study type .................................................................. 17**

**Table S5. Sensitivity analysis comparing DL and REML models: Meta-analysis results for cognitive impairment subtypes by study design .......................................................... 17**

**Table S6. Sensitivity analysis comparing DL and REML models: Subgroup analysis for cognitive impairment by duration of diabetes ................................................................... 18**

**Table S7. Sensitivity analysis comparing DL and REML models: Subgroup analysis for cognitive impairment by follow-up duration ..................................................................... 18**

**Table S8. Sensitivity analysis comparing DL and REML models: Subgroup analysis for cognitive impairment by cognitive assessment tools ......................................................... 19**

**Table S9. The pooled effect size by trim-and-fill method in cohort studies .................... 19**

**Table S10. PRISMA Checklist ............................................................................................ 20**

**Supplement Figure legends**

**Figure S1. Sensitivity analysis results for cross-sectional studies (fixed model)..............24**

**Figure S2. Leave-one-out sensitivity analysis for cross-sectional studies ........................24**

**Figure S3. Sensitivity analysis results for cohort studies (random model)...................... 25**

**Figure S4. Leave-one-out sensitivity analysis for cohort studies ......................................25**

**Figure S5. Funnel plot of standard error of log OR for the association of diabetic retinopathy and cognitive impairment ................................................................................26**

**Figure S6. Enhanced funnel plot of standard error of log OR for the association of diabetic retinopathy and cognitive impairment .................................................................26**

**Supplementary methods**

Search strategy

Dates: 01.01.2000- 01.03.2025

Search Terms

**Population:** Diabetes Mellitus, Type 2[Mesh], T2D, T2DM, Type 2, diabetes Type 2 Diabetes Mellitus, NIDDM, non insulin dependent diabetes, Diabetic Patient, Adult-Onset Diabetes Mellitus,

**Exposure/Intervention:** Diabetic Retinopathy[Mesh], DR, Diabetic Macular Edema, DME, Retinopath*, Diabetic Eye Disease, Fundus Ophthalmoscopy, Retinal Photography,

**Outcome:** Cognition Disorders[Mesh], Cognitive Dysfunction[Mesh], Cognitive Decline, Cognitive Impairment, Dementia[Mesh], Alzheimer's Disease[Mesh], Vascular Dementia, Mild Cognitive Impairment, MCI, Cognitive Defect, Memory Disorder, Executive Function, Cognitive Test, MMSE, MoCA,

**Study type:** Cohort Studies, Prospective Studies[Mesh], Retrospective Studies[Mesh], Observational Study, Longitudinal Studies, Epidemiological Studies, Follow-Up Studies, real world, real life, RWE, database, registry, NOT Clinical Trial [Publication Type], NOT Review [Publication Type], NOT Meta-Analysis [Publication Type], NOT Case Report [Publication Type], NOT Editorial [Publication Type], NOT Letter [Publication Type].

**Using PubMed as an Example**

#1 ("Diabetes Mellitus, Type 2"[Mesh]) OR (T2D[Title/Abstract]) OR (T2DM[Title/Abstract]) OR ("Type 2"[Title/Abstract] AND diabet*[Title/Abstract]) OR (NIDDM[Title/Abstract]) OR ("non insulin dependent diabetes"[Title/Abstract]) OR ("Diabetic Patient"[Title/Abstract]) OR ("Adult-Onset Diabetes Mellitus"[Title/Abstract])

#2 ("Diabetic Retinopathy"[Mesh]) OR (DR[Title/Abstract]) OR ("Diabetic Macular Edema"[Title/Abstract]) OR (DME[Title/Abstract]) OR (Retinopath*[Title/Abstract]) OR ("Diabetic Eye Disease"[Title/Abstract]) OR ("Fundus Ophthalmoscopy"[Title/Abstract]) OR ("Retinal Photography"[Title/Abstract])

#3 ("Cognition Disorders"[Mesh]) OR ("Cognitive Dysfunction"[Mesh]) OR ("Cognitive Decline"[Title/Abstract]) OR ("Cognitive Impairment"[Title/Abstract]) OR ("Dementia"[Mesh]) OR ("Alzheimer's Disease"[Mesh]) OR ("Vascular Dementia"[Title/Abstract]) OR ("Mild Cognitive Impairment"[Title/Abstract]) OR (MCI[Title/Abstract]) OR ("Cognitive Defect"[Title/Abstract]) OR ("Memory Disorder"[Title/Abstract]) OR ("Executive Function"[Title/Abstract]) OR ("Cognitive Test"[Title/Abstract]) OR (MMSE[Title/Abstract]) OR (MoCA[Title/Abstract])

#4 ("Cohort Studies"[Mesh]) OR ("Prospective Studies"[Mesh]) OR ("Retrospective Studies"[Mesh]) OR ("Observational Study"[Title/Abstract]) OR ("Longitudinal Studies"[Title/Abstract]) OR ("Epidemiological Studies"[Title/Abstract]) OR ("Follow-Up Studies"[Title/Abstract]) OR ("real world"[Title/Abstract]) OR ("real life"[Title/Abstract]) OR (RWE[Title/Abstract]) OR (database[Title/Abstract]) OR (registry[Title/Abstract])

#5 #1 AND #2 AND #3 AND #4

#6 "Clinical Trial" [Publication Type]

#7 "Review" [Publication Type]

#8 "Meta-Analysis" [Publication Type]

#9 "Case Reports" [Publication Type] OR "Editorial" [Publication Type] OR "Letter" [Publication Type]

#10 #5 NOT (#6 OR #7 OR #8 OR #9)

**Table S1. Characteristics of the studies included in the review.**

| Study ID | Study design | Country | Patients | Age  (Years) | Duration of DM (Years) | Age of Onset of DM | HbA1c（%） | Sample size（% males） | Sample（N/C） | Follow up (Years) | DR identification | Cognitive measurements | OR（95%Cl） | Adjustment factors |
| --- | --- | --- | --- | --- | --- | --- | --- | --- | --- | --- | --- | --- | --- | --- |
| Oğuz 2009^[21]^ | case-control study | Turkey | Type-2 diabetic patients from Family Medicine, Neurology, and Ophthalmology clinics of Fatih University Hospital in Ankara | 57.0±8.1 | 8.3±5.9 | / | 7.6±1.2 | 75 (46.7%) | 75/49 | / | Ophthalmologic exam (BDR, PPDR, PDR, maculopathy) | MMSE | Registration scores were negatively correlated with DR (P = 0.015). | Age, educational status, HTN, nephropathy, ischemic heart disease, peripheral neuropathy, and HbA1c levels. |
| Naidu2016^[22]^ | case-control study | United Kingdom | Primary care cohort (South London Diabetes Study, SOUL-D) with newly diagnosed T2DM (within 6 months) | <50 years: 26.1% (cases), 32.4% (controls) 50–59 years: 29.0% (cases), 44.1% (controls) 60–69 years: 23.2% (cases), 14.7% (controls) ≥70 years: 20.3% (cases), 8.8% (controls) | Newly diagnosed (<6 months). | / | Cases: 7.2 ± 1.6% Controls: 6.9 ± 1.1% (P = 0.264) | 137 (cases: 44.9% ; controls: 64.7% ) | Cases (cognitive impairment, TICSM score ≤17): 69 Controls (randomly selected): 68 | / | Defined as any retinal hemorrhage or microaneurysm in either eye, graded by an ophthalmologist. Retinal Imaging: Stereoscopic photographs analyzed by SIVA for vessel caliber, arterio-venous ratio, fractal dimension, and tortuosity. | Modified Telephone TICSM. Cognitive impairment defined as lowest 10% of TICSM scores (≤17). | Retinopathy prevalence: 22.4% (cases) vs. 11.9% (controls), P = 0.11. Venular tortuosity: Significantly higher in cases (mean difference = 0.012, P = 0.013), persisting after adjustment for covariates (B = 0.013, 95% CI 0.002–0.025, P < 0.05). | Demographic: Age, sex, ethnicity (white/black/Asian). Clinical: DN, BMI, SBP/DBP, antihypertensive medication. Metabolic: HbA1c. |
| Lu2019^[23]^ | case-control study | China | Renmin Hospital, Wuhan, China | DR group: 61.19 ± 8.41 years  T2DM group: 61.04 ± 7.54 years  HC group: 59.81 ± 3.68 years | >15 but <30 | / | DR group: 8.98 ± 2.24% T2DM group: 7.98 ± 0.89% HC group: 5.31 ± 0.40% | Total: 83 participants (53 T2DM patients + 30 healthy controls) DR group: 26 (14 male / 12 female) T2DM group: 27 (10 male / 17 female) HC group: 30 (17 male / 13 female) | DR group: 26 T2DM group: 27 HC group: 30 | / | Fundus examination (microaneurysms and/or small hemorrhage as diagnostic criteria) | MoCA | Chi-square analysis showed a correlation between DR and DCI (χ² = 4.6, df = 1, p = 0.032). | Age, gender, education level, BMI, SBP, DBP, FPG, HbA1c, TC, TG, HDL-C, LDL-C. |
| Maimaitituerxun2024^[24]^ | case-control study | China | Endocrinology Department of Xiangya Hospital affiliated to Central South University | N：60.2±10.3  C：60.4±9.5 | 53.8% had DM ≥10 yrs | / | 67.7% had HbA1c >7% | 1001 (61.2%) | 705/296 | / | Medical records | MMSE (Pearson criteria) | Adjusted OR = 1.55 (95% CI: 1.02–2.35), p = 0.042 | Age, sex, marital status, educational level, household income, location of residence, primary caregiver, current work status, smoking, drinking, PA, BMI, HbA1c, DM duration , family history of DM, stroke, HTN, CHD, fatty liver, DN, diabetic foot. |
| Umegaki2012^[25]^ | cohort study | Japan | Japanese Elderly Diabetes Intervention Trial (J-EDIT) | 70.6±4.3 | / | / | 8.0±0.8 | 261 (42.5%) | Cognitive decline group (≥5-point MMSE decline): 23 Cognitively preserved group: 238 | 6 years | Fundoscopic examination (classified as mild or serious DR) | MMSE | Unadjusted: OR = 2.02 (95% CI: 0.81–5.07), P = 0.133 (retinopathy presence) Adjusted (Model 2, including TG): OR = 2.78 (95% CI: 0.96–8.08), P = 0.060 Adjusted (Model 4, including TG): OR = 2.08 (95% CI: 0.68–6.33), P = 0.198 | Age, sex, GDS-15, SBP, TG, HDL-C, DN, neuropathy, and other metabolic factors (e.g., BMI, HbA1c, albumin). |
| Bruce2014^[26]^ | cohort study | Australia | Fremantle Diabetes Study (FDS), Western Australia | Baseline: 57.5±9.2  Follow-up: 72.2± 9.1 | At baseline: Median 2.8 (IQR: 0.7–6.0) At follow-up: ~17.8 years | 52.7 ± 9.4 years (baseline) | Median 6.9 (IQR: 6.0–8.4) | 335 (50.7%) | Cognitively normal: 276 Cognitive impairment: 27 Dementia: 17 Unclassifiable: 15 | 14.7 ± 1.1 years | Retinal photography (non-mydriatic camera) or ophthalmoscopy; classified as "any retinopathy." | Screening: MMSE Definitive: CDR | Cognitive impairment + dementia combined: OR = 3.11 (95% CI: 1.15–8.38) Cognitive impairment alone: Not significant (DR not retained in the final model) Dementia alone: Not significant (DR not retained in the final model) | Age, sex, education, smoking status, fasting glucose, insulin therapy, BMI, BP, lipid levels, diabetic complications (nephropathy, neuropathy), and cardiovascular comorbidities (stroke/TIA, ischemic heart disease). |
| Trento2017^[27]^ | cohort study | Italy | Urban diabetes clinics | N: 66.4±6.9  C: 66.3±8.2 | / | / | N: 8.30±1.2  C: 7.87±1.03 | 498 (not explicitly stated) | 181/98 | 8 years | ETDRS classification via Canon CR6-45NM fundus camera (2-field, 45° digital color photography) | MMSE | OR 0.90 per MMSE score point (95% CI: 0.83-0.99, p=0.025) | Age, sex, insulin treatment, HbA1c (stratified by baseline DR status), MMSE score |
| de Bresser2010^[28]^ | cohort study | The Netherlands | General practitioners (general population) | 65.6±5.6 | 9.1±6.3 | / | 6.9±1.1 | 68 (47%) | 11 with retinopathy, 24 with neuropathy, 8 with albuminuria | 4 years | Single-field retinal photography (Zeiss FF 450) centered on the macula, scored for presence/absence of non-proliferative or worse retinopathy by an ophthalmologist | Composite score derived from 11 neuropsychological tasks covering 5 domains (attention/executive functions, processing speed, memory, abstract reasoning, visuoconstruction); changes measured using RBI | DR was not significantly associated with cognitive decline (standardized beta-coefficient for composite RBI: 0.08, 95% CI: -0.22 to 0.38, p > 0.01). | Age, sex, IQ (for cognition); additional adjustments for mean arterial pressure, BP-lowering drugs, and baseline brain infarcts in secondary analyses. |
| Yen2024^[29]^ | cohort study | Taiwan | National Health Insurance Research Database (NHIRD) | ≥50 | Newly diagnosed T2D (2009–2014) | / | / | 1,212,966 (not explicitly stated; match) | Without microvascular disease: 568,577 With microvascular disease: 79,241 (DKD: 24,790; DR: 8,316; DN: 37,527) | 6.54 years | ICD-9/10-CM codes (diagnosed within 1 year of T2DM diagnosis) | Dementia outcomes identified via ICD codes (AD, VD, other dementia) and medication records | DR vs. no microvascular disease: AD: aHR 1.02 (0.78–1.33) VD: aHR 1.05 (0.79–1.39) Other dementia: aHR 0.94 (0.81–1.09) ACD: aHR 0.97 (0.86–1.11) | Propensity score matching for demographics, comorbidities (e.g., HTN, dyslipidemia, stroke), and medications (e.g., antidiabetic drugs, statins). Adjusted for sex, age, obesity, smoking, alcohol-related disorders, comorbidities, and medications in multivariable models. |
| Ravazzani2022^[30]^ | cohort study | Brazil | Tertiary hospital in southern Brazil | 61.1±9.8 | 12.6±8.9 | / | 7.6±4.2 | 251 (43.4%) | Baseline: 251 patients Follow-up: 134 patients (56.6% female) | 18.4±5.0 | Retinal mapping under drug-induced mydriasis (indirect binocular ophthalmoscopy, slit-lamp biomicroscopy, fluorescein angiography, and optical coherence tomography). Classified as absent, non-proliferative DR, proliferative DR, or macular edema. | GCS(z): Composite of MMSE, Semantic Verbal Fluency Test, Trail Making Test A/B, and Word Memory Tests (CERAD). | Baseline: OR 2.50 (1.18–5.34) for GCS(z) < 0. Follow-up: DR was not a significant predictor (not retained in the final multivariate model). | Baseline model: Adjusted for age ≥ 65 years, schooling ≤ 6 years, arterial HTN, depression symptoms (PHQ-9 > 9), DM duration ≥ 10 years, PA, and CVD. Follow-up model: Adjusted for schooling ≤ 6 years, DM duration ≥ 10 years, depression symptoms, arterial HTN, and CVD. |
| Tlemçani2022^[31]^ | cohort study | Morocco | Endocrinology consultation of Sheikh Khalifa Ibn Zaid Hospital, Casablanca | Median 65 (IQR: 59-70) | Median 15 (IQR: 9-20) | / | Median 8 (IQR: 7.1-9.13) | 100  (65%) | 52/47 | 1 year | DR was observed in patients who reported the occurrence of proliferative DR at baseline and underwent standardized eye examinations. | MMSE adapted to the Maghrebi population (MMSE <27/30 defined cognitive impairment). | DR was significantly associated with cognitive decline (coefficient β: -1.825; 95% CI: -3.109 to -0.541; p-value <0.001). | Age, dyslipidemia, diabetic retinopathy, creatinine (in multivariate analysis). |
| Doney2022^[32]^ | cohort study | United Kingdom | GoDARTS bioresource (Tayside region, Scotland) | Median 68.4 (IQR: 60.1–75.5) | Median 7.1 (IQR: 3.8–11.8) | / | 7.52±1.11 | 6,111 (56%) | Incident dementia cases: 635 (ACD: 635; AD: 327; VD: 218) Non-dementia: 5,476 | Median 9.8 years (IQR: 5.7–10.6) | RVMs from DR screening photographs using VAMPIRE software (central retinal arteriole/venule equivalent [CRAE/CRVE], tortuosity [TORTA/V], fractal dimension [FDA/V]). | EMR adjudicated for dementia diagnosis (ACD, AD, VD). | ACD: Increased arteriolar fractal dimension (FDA) (csHR 1.17; 1.08–1.26); wider arteriolar calibre (CRAE) (csHR 0.9; 0.83–0.98). AD: Increased FDA (csHR 1.33; 1.16–1.52); reduced venular fractal dimension (FDV) (csHR 0.85; 0.74–0.96); wider venular calibre (CRVE) (csHR 0.87; 0.78–0.97). VD: Increased FDV (csHR 1.22; 1.07–1.40). | Age, sex, DM duration, smoking, cardiovascular disease history, BMI, SBP, TC, HDL, HbA1c, ApoE4 genotype. |
| Lee2023^[33]^ | cohort study | United States | Adult Changes in Thought (ACT) cohort, Kaiser Permanente Washington (KPW) | Median 75 (IQR: 71–80) | diabetes medication fills≥2x/year | / | ＞5 years | 536 participants (46% male) | DR >5 years: 177 DR≤5 years: 92 No DR: 267 | Median 5.9 years (IQR: 3.2–9.0) | Clinical diagnosis via ICD-9/10 codes (95% confirmed by ophthalmologists/optometrists). | Screening: CASI. Diagnosis: Expert consensus using DSM-IV criteria for dementia and NINCDS-ADRDA for AD. | All-cause dementia: DR >5 years: HR 1.81 (1.23–2.65), p=0.003 (Model A); adjusted HR 1.69 (1.14–2.50), p=0.01 (Model B). Alzheimer’s dementia: DR >5 years: HR 1.80 (1.15–2.82), p=0.01 (Model A); adjusted HR 1.73 (1.10–2.74), p=0.02 (Model B). | Model A: Age, sex, race, education, APOE ε4, smoking, ACT cohort, glaucoma/AMD duration. Model B: Model A + microalbuminuria (ACR), long-term glycemia, eGFR constructs (mean, trajectory, variability). Model C: Model B + vascular diseases (heart disease, cerebrovascular disease, CHF, HTN). |
| Roberts2014^[34]^ | cohort study | USA | Olmsted County, Minnesota residents (population-based study) | Median 79.3 (IQR: 74.9–83.4) | Median 7.94 (stratified into ≤7.94 vs. >7.94 ) | Early onset: ≤65 years  Late onset: >65 years | <7%: 183 subjects (28.4% MCI incidence) ≥7%: 47 subjects (38.3% MCI incidence) | 1,450 (49.8%) | 248/1202 | Median 4.0 years (IQR: 2.5–5.1) | DR was defined by medical record review (ICD codes not specified). | Clinical Dementia Rating scale, neurological evaluation, neuropsychological testing (assessing memory, executive function, language, visuospatial skills). MCI subtypes: amnestic (aMCI) and non-amnestic (naMCI), single-domain (SD) vs. multiple-domain (MD). | Retinopathy: HR = 1.77 (1.02–3.05) for any MCI (Model 1),P=0.04; adjusted HR =1.92 (1.05–3.49), P=0.03(Model 2)；adjusted HR = 2.37 (1.28–4.39), P=0.006 in Model 3 (stroke excluded). | Model 1: Adjusted for sex and education (≤12 vs. >12 years). Model 2: Added APOE ε4 genotype, HTN, obesity, depression, statin use, exercise, CHD, dyslipidemia, and stroke. Model 3: Excluded subjects with stroke history (n = 108). |
| Hendrie2018^[35]^ | cohort study | USA | Eskenazi Health Services (urban health care system in Indianapolis, IN) | Caucasians:  N: 64.6±6.8  C: 62.3±6.7  African Americans:  N: 64.5±6.2  C: 61.8±6.3 | Caucasians:  10.3 (N)  12.7 (C) African Americans: 11.6 (N)  14.2 (C) | / | / | 5,228 Caucasians: 33.4%  African Americans: 29.8% | Caucasians: 2,403 (no dementia: 2,021; dementia: 382) African Americans: 2,825 (no dementia: 2,294; dementia: 531) | Caucasians: Median 12.2 years (IQR: 8.5–16.5) African Americans: Median 13.8 years (IQR: 9.3–17.8) | DR was identified via ICD-9 codes. | Dementia diagnoses based on ICD-9 codes (290., 294., 331.*, etc.) from EMR. | DR rates: Higher in African Americans (36.8% no dementia, 39.4% dementia) vs. Caucasians (27.3% no dementia, 23.4% dementia), but no direct OR for dementia association provided. Hypoglycemia: Associated with dementia in both groups (Caucasians: OR not reported, p=0.0241; African Americans: OR not reported, p<0.0001). | Adjusted for baseline age, gender, BMI, and comorbidities (e.g., cerebrovascular disease, HTN, depression). Sensitivity analyses excluded participants with cerebrovascular disease or hypoglycemia. |
| Yu Ji Hee2020^[36]^ | cohort study | Korea | NHIS database (nationwide) | N: 72.2±7.3 (N)  C: 59.1±10.3 (C） | ≥5 years  51.8% (N)  35.6% (C) | / | / | 1,917,702 N: 38.7%  C: 58.9% | 92,758 /1,824,944 | 5.1 years | ICD-10 code H36.0 | ICD-10 codes (F00, F01, F02, F03, G30, G31) + anti-dementia medications | Diabetic retinopathy HR: 1.09 (1.07–1.11) for all dementia; 1.10 (1.07–1.12) for AD; 1.08 (1.03–1.14) for VD | Age, sex, smoking, alcohol, exercise, income, glucose, diabetes duration, BMI, dyslipidemia, HTN, CKD, stroke, IHD, depression, OHAs, insulin |
| Hugenschmidt2014^[37]^ | cohort study | USA | ACCORD trial participants (multicenter) | 62.3±5.7 | 9.7±6.8 | / | 8.3±1.0 | 1,862 (56.1%) | 432/1430 | 40 months | ETDRS severity scale (levels 10–47), categorized as: none, mild NPDR, moderate/severe NPDR/PDR | MMSE, DSST, RAVLT, Stroop test | DSST decline: Moderate/severe DR vs. none: β = −2.89 (−3.67 to −2.12), P = 0.01 (Model 2). MMSE decline: Moderate/severe DR vs. none: β = −0.42 (−0.64 to −0.20), P = 0.04 (Model 2). | Model 1: Age, sex, ethnicity, education, smoking, geographic region, treatment assignment. Model 2: Model 1 + diabetes duration, HbA1c, HDL, triglycerides, SBP, antihypertensive meds, depression, alcohol, neuropathy, visual acuity. |
| Exalto2014^[38]^ | cohort study | USA | Kaiser Permanente Northern California Diabetes Registry | 70.6±6.8 | 11.6±9.5 | / | 7.8±1.8 | 29,961 (54.0%) | 2,008/27,953 | 6.6 years | Severe DR (proliferative DR or macular edema) via ICD-9 codes or treatment records (CTP4 codes) | Dementia diagnosis via ICD-9 codes (290.0, 331.0, 290.4x, 290.1) from medical records | Demographics-adjusted HR: 1.42 (1.27–1.58) Fully-adjusted HR: 1.32 (1.17–1.49) (see notes for details) | Model 1: Age (time scale). Model 2: Age, sex, race, education. Full model: Model 2 + medical utilization, diabetes composite (duration, HbA1c, insulin, hypoglycemia), vascular composite (CVD, HTN, etc.), BMI, smoking. |
| Zhong2023^[39]^ | cohort study | UK and China | UK Biobank (UKB) and Guangzhou Diabetic Eye Study (GDES) | UKB: 59.3±7.3  GDES: 64.5±7.6 | Median 7.0 (IQR： 3.0–12.0) | / | UKB: 7.0±1.3 GDES: 6.9±1.3 | UKB: 27,773 (59.5%) GDES: 1,307 (42.2%) | UKB: 27,773  GDES: 1,307 | UKB: Median 12.0 years (IQR 11.1–12.8)  GDES: Median 2.8 years (IQR 2.1–3.1) | UKB: DR events identified using ICD-10 and OPCS4 codes. GDES: DR graded according to the ETDRS scale; OCTA used for retinal capillary density assessment. | UKB: Brain MRI (gray/white matter volume, hippocampus volume), prospective memory, and reaction time tests. GDES: GCS based on orientation, attention, episodic memory, and visuospatial abilities. | UKB: 1-SD decrease in gray matter volume: HR 1.51 (1.11, 2.06) for DR. Impaired reaction time: HR 1.31 (1.11, 1.57) for DR. GDES: Lowest GCS tertile vs. highest: HR 1.78 (1.19, 2.68) for referable DR. | UKB: Age, sex, ethnicity, education, income, smoking, PA, BMI, BP, HbA1c, lipids, medications, ApoE4 status, visual impairment. GDES: Age, sex, SBP, MAP, HbA1c, DM duration, baseline DR. |
| Liu2017^[40]^ | cross-sectional study | China | Fuzhou General Hospital, Nanjing Military Command | DR: 63.9 ± 6.9; NDR: 59.8 ± 9.3; NC: 58.6 ± 6.9 | / | / | DR: 9.56 ± 1.58; NDR: 8.16 ± 1.69; NC: 5.77 ± 0.21 | DR:15 (46.7%); NDR:17 (52.9%); NC:21 (42.9%) | DR: 15; NDR: 17; NC: 21 | / | 2014 Chinese Clinical Guidelines for DR Diagnosis and Treatment | MMSE, MoCA, TMT-A, Wechsler Memory Scale (Picture Recall) | Cognitive function in DR group was significantly lower than in NDR and NC groups | Sex, age, DBP, education level, TC, TG, LDL-C, HDL-C, creatinine |
| Su2021^[41]^ | cross-sectional study | China | Dept. of Endocrinology, Shaanxi Provincial People's Hospital | 40-70 | 11.22 ± 5.60 | / | 9.04 ± 2.44 | 113 (76.1%) | 113 / 72 | / | Fundoscopy indicating retinopathy | MMSE, MoCA, CDT, TMT-A/B | / | Age, sex, education level, BP, blood lipids, etc. |
| Wu2022^[42]^ | cross-sectional study | China | Dept. of Ophthalmology, Gansu Provincial Hospital of TCM | 39-80 | 5-22 (Median) | / | 8.06 - 9.39 (Group means) | 159 (64.2%) | NDR: 57; PDR: 15 | / | 2019 AAO Guidelines; Fundus photography | MMSE, MoCA | Negative correlation (P < 0.05) | Age, sex, DM duration, HbA1c, BP, blood lipids, etc. |
| Xiao2023^[43]^ | cross-sectional study | China | Inpatients, Dept. of Endocrinology, Affiliated Hospital of Hubei University of Medicine | Median: Normal Cognition 57, MCI 62 | Median: Normal Cognition 7, MCI 5 | / | Normal Cognition: 8.5%; MCI: 9.7% | 344 (54.4%) | Normal Cognition: 203; MCI: 141 | / | International Clinical Classification Scale (NDR/NPDR/PDR); Fundus photography | MMSE screening followed by MoCA (<26 for MCI) | DR was significantly associated with cognitive impairment (P < 0.05) | Age, education level, BMI, BP, HbA1c, blood lipids (TC, TG, HDL-C, LDL-C), uric acid |
| Yang2024^[44]^ | cross-sectional study | China | Inpatients, Dept. of Endocrinology, Shanxi Provincial People's Hospital | 28-80 | Median: Non-MCI 10, MCI 15 | / | Median: Non-MCI 8.0, MCI 8.2 | 212 (67.9%) | MCI: 145; Non-MCI: 67 | / | International Clinical Classification Scale (2002); Fundus photography | ADL, MMSE, MoCA | OR = 16.2 (95% CI: 3.586–73.280) | Age, education level, DM duration, sex, BMI, HTN, HbA1c |
| Zhu2019^[45]^ | cross-sectional study | China | Newly diagnosed, untreated inpatients, Dept. of Endocrinology, Yancheng Third People's Hospital | NC: 54.70 ± 4.70; NDR: 53.80 ± 5.60; DR: 55.60 ± 4.20 | / | / | NDR: 8.84 ± 1.64; DR: 9.02 ± 1.74; NC: 5.25 ± 0.45 | 97 (56.5%) | DR: 39; NDR: 30; NC: 28 | / | Fundus fluorescein angiography results (DR vs. NDR) | MMSE,TMT-A, TMT-B | For each 1 mmol/L increase in FBG: OR=1.353 for lower MMSE (95% CI: 1.034–1.771, P=0.028). For each 1% increase in HbA1c: OR=1.362 for lower MMSE (95% CI: 1.030–1.801, P=0.030). | Sex, age, BMI, BP, blood lipids (TG, TC, HDL-C, LDL-C) |
| Liu2016^[46]^ | cross-sectional study | China | Inpatients/outpatients, Dept. of Endocrinology, Taishan Medical College Affiliated Hospital; healthy volunteers | T2DM: 56.9 (M10, F8); T2DR: 56.7 (M11, F8); NC: 52.1 (M12, F9) | T2DM: 7.86 ± 3.16; T2DR: 11.04 ± 7.23 | / | T2DM: 8.28 ± 1.63; T2DR: 8.26 ± 1.77; NC: Not tested | 58 (56.9%) | T2DR: 19; T2DM: 18; NC: 21 | / | 2015 ADA Standards of Medical Care; dilated ophthalmoscopy and fundus photography; exclusion of other causes | MMSE (all subjects ≥27, excluding clinical cognitive impairment) | T2DR group showed decreased ALFF in brain regions (bilateral posterior cerebellum, left precuneus, left superior frontal gyrus, etc.) compared to T2DM group, suggesting impaired neural activity. For each 1% increase in HbA1c: OR=1.362 for lower MMSE (95% CI: 1.030–1.801, P=0.030). | Baseline: Age, sex, education (≥6 years), right-handedness. Clinical: FPG, blood lipids (LDL-C, HDL-C, TG, CHOL), BP. |
| Finger2014^[47]^ | cross-sectional study | Australia | Royal Victorian Eye and Ear Hospital clinics; community-dwelling | 66 ± 11 | 14 ± 10 | / | 7.74 ± 1.52 | 501 (65%) | No cognitive impairment (n=450), Cognitive impairment (n=51) | / | Dilated fundus photography (Canon CR6-45NM); graded using modified Airlie House Classification System | 6-CIT/ Short Orientation-Memory-Concentration Test | Not significant (P=0.471 for DR severity; no OR reported) | Age, sex, education level, living alone, DM duration, insulin use, SBP and DBP, HbA1c, BMI, waist-to-hip ratio, previous stroke, depression (HADS score), PA |
| Moran2016^[48]^ | cross-sectional study | Australia | Southern Tasmania (postcodes 7000-7199) | T2DM: 67.3 ± 6.7; Control: 72.9 ± 6.7 | Median: 6 years (IQR 4–11) | 57.6 ± 10.9 | T2DM: 7.1 ± 1.2; Control: 5.6 ± 0.3 | 451 (T2DM: 59%; Control: 54%) | 270 (T2DM) / 181 (Control) | / | Simplified Wisconsin grading system; Canon CR-DGi non-mydriatic camera; graded by expert. DR defined by microaneurysm, hemorrhage, hard exudates, etc. | MRI-based brain volume (gray/white matter, hippocampus). No specific cognitive test mentioned. | Model 1 (Unadjusted): 1.00 (0.68 to 1.47) Model 2 (Adjusted for age, sex): 1.47 (0.96 to 2.26) Model 3 (Adjusted for age, sex, vascular risks*): 1.41 (0.85 to 2.32) | Model 1: Unadjusted. Model 2: Age, sex. Model 3: Age, sex, vascular risk factors* (smoking history, HTN, SBP and DBP, BMI, history of stroke, IHD, hyperlipidaemia). *Specific list from source. |
| Sanahuja2016^[49]^ | cross-sectional study | Spain | Outpatient clinic at University Hospital Arnau de Vilanova, Lleida, Spain | Median: 61 years (Q1–Q3: 54–68)/Median: 59 years (Q1–Q3: 48–66) | Median: 11 years (Q1–Q3: 6–20)/Median: 6 years (Q1–Q3: 2–10) | / | Median: 8.1(Q1–Q3: 7.2–9.1)/Median: 7.1 (Q1–Q3: 6.5–7.9) | 312 (51% male) | 153/159 | / | Graded by an experienced ophthalmologist using international consensus criteria: 1. Mild nonproliferative DR (microaneurysms only) 2. Moderate nonproliferative DR 3. Severe nonproliferative DR 4. Proliferative DR (neovascularization/vitreous hemorrhage). | MRI-based assessment of white WMLs and lacunar infarcts. No specific cognitive test mentioned. | Presence of DR associated with higher SVD burden: - SVD prevalence: OR = 2.65 (95% CI 1.25–5.63) for high SVD score. - Severity of DR correlated with SVD severity (P<0.01). | Age, SBP/DBP, HTN, carotid plaques, microalbuminuria, HbA1c, and antidiabetic/antiplatelet treatments. |
| Gorska2014^[50]^ | cross-sectional study | Poland | Outpatient clinic at the Department of Internal Medicine and Diabetology, University Hospital No. 1 in Lodz, Poland | 73.6 ± 4.8 | 8.69 ± 6.23 | / | 7.24 ± 0.68 | 276 (46.0% male) | MCI group: 87 Non-MCI group: 189 Depressive syndrome group: 82 Non-depressive syndrome group: 194 MCI and depressive syndrome group: 25 Non-MCI and non-depressive syndrome group: 251 | / | DR was assessed based on the existence of nephropathy, retinopathy, neuropathy, cardiovascular disease (CVD), and stroke. | MoCA | Univariate logistic regression model：OR = 2.25 (1.7–2.96)  Multivariate logistic regression model：OR = 1.63 (1.07–2.45)，P=0.021 | Demographic: Gender, age, education, marital status Lifestyle: Smoking status, PA Clinical: BMI, HbA1c, lipid levels, diabetes treatment, micro- and macrovascular complications, HTN, hyperlipidemia, comorbidities, history of hypoglycemia Biochemical: TC, LDL, HDL, TG |
| Gorska2016^[51]^ | cross-sectional study | Poland | Outpatient clinic, Department of Internal Medicine and Diabetology, University Hospital No. 1 in Lodz | 74.7 ± 3.9 (MCI group) / 72.5 ± 4.9 (non-MCI group) | N: 10.63 ± 6.2  C: 6.45 ± 5.07 | / | N: 7.62 ± 0.69 C: 7.40 ± 5.5 | 194 (51.6% female in MCI group; 37.8% female in non-MCI group) | MCI: 62 Non-MCI: 132 | / | Medical history/complications | MoCA | DR & MCI: OR = 2.16 (1.56–2.99) (univariate model) DR was not retained in the multivariable model. | Demographic: Age, gender, education Clinical: BMI, DM duration, HbA1c, CVD, HTN, hyperlipidemia, nephropathy, hypoglycemia, comorbidities Biochemical: TC,TG, LDL, HDL, adiponectin, leptin, IL-1β Treatment: Insulin, oral antidiabetic drugs |
| Yu2020^[52]^ | cross-sectional study | China | Endocrinology Department, The First Clinical Hospital of Harbin Medical University | MCI group: 57.50 (48.50–62.75) Normal cognition group: 55.50 (50.25–63.00) | N: 13.00 (5.00–17.00) years C: 10.00 (7.00–15.75) years | / | N: 9.42 ± 2.16 C: 8.39 ± 1.45 | 124 (57.3% male in normal cognition group; 60.7% male in MCI group) | MCI: 56/68 | / | Diagnosed via FFA; classified as NPDR or PDR | MMSE; MCI defined as MMSE score <27 and >23 | Serum NSE (neuron-specific enolase) was independently associated with MCI (OR: 1.606, 95% CI: 1.264–2.041, P<0.001) after full adjustment. | Model 1: Unadjusted Model 2: Age, sex Model 3: Age, sex, DM duration, education level, HbA1c, BMI, TC, fasting C-peptide, UA Model 4: Model 3 + lifestyle factors (smoking, alcohol), HTN, diabetic complications (dn, peripheral neuropathy), and treatments (insulin, metformin, statins) |
| Sajeev2023^[53]^ | cross-sectional study | India | Ophthalmology outpatient services of a tertiary hospital | DR+ group: 59.90 ± 10.44 years; DR- group: 53.25 ± 11.14 years | / | / | / | 171 (63.2% male in DR+ group, 55.7% male in DR- group) | DR+ (n = 83), DR- (n = 88) | / | Early Treatment Diabetic Retinopathy Study (ETDRS) criteria | MoCA | / | Age, DM duration, HTN, education level, visual acuity, comorbid mental/neurocognitive disorders |
| Yokoyama2014^[54]^ | cross-sectional study | Japan | Outpatients from Jiyugaoka Internal Medicine Outpatient Clinic | 68 ± 8 | 13 ± 9 | / | 6.8 ± 0.8 | 1,449 (64.8% ) | Non-diabetes group: 126 Diabetes group: 1,323 | / | DR was diagnosed after pupillary dilation by ophthalmologists. | MMSE, with abnormal cognition defined as MMSE score <24. | Unadjusted OR: 1.99 (1.23–3.22), P<0.01  Adjusted OR (Model 2): 1.82 (1.04–3.19), P<0.05 | Model 1: Age, sex, BMI, current smoker. Model 2: Age, sex, BMI, current smoker, DM duration, HTN, dyslipidemia, history of CHD, and stroke. |
| Tekin2009^[55]^ | cross-sectional study | Turkey | Family Medicine, Neurology, and Ophthalmology clinics of Fatih University Hospital, Ankara | 57 ± 8.1/54.7 ± 8.46 | 8.3 ± 5.9 | / | Group 1 (HbA1c ≤ 6%): n = 8 Group 2 (HbA1c > 6.1%): n = 67 | Diabetic group: 75 (46.7% male) Control group: 49 (59.2% male) | 75/49 | / | Ophthalmologic examination (fundus changes categorized as background, preproliferative, proliferative, maculopathy) | MMSE | Logistic regression showed diabetic group was 7.262 times (95% CI: 2.324–22.698) more likely to have cognitive impairment (MMSE ≤ 24) compared to controls. Negative correlation between DR and registration scores (P = 0.006) | Age, educational level, HTN, DN, ICD, peripheral neuropathy. |
| Tong2014^[56]^ | cross-sectional study | China | Affiliated Hospital of Jining Medical University, Shandong, China | 51.2 ± 5.7 | 9.5 ± 3.0 | / | 8.2 ± 1.5 | 89 (41.6%) | 29 (DR group) / 30 (DM group) / 30 (NC group) | / | Diagnosed by fundoscopy after pharmacologic pupillary dilation; minimum criterion was the presence of at least one microaneurysm. | Not explicitly mentioned (study focused on brain metabolite changes via ¹H-MRS, not cognitive testing). | N/A | Age, gender, BP, FPG, HbA1c, exclusion of cerebrovascular/neurological diseases, HTN, liver/kidney dysfunction, alcohol/drug abuse. |
| Gao2016^[57]^ | cross-sectional study | China | Community-dwelling elderly residents in Tianjin, China | 72.4 ± 3.5 | 11.2 ± 3.7 | Diagnosed in the 3rd or later decades of life (specific mean not provided) | 7.21 ± 1.34 (total population), 7.73 ± 1.12 (MCI subjects), 6.71 ± 1.45 (cognitive intact subjects) | 8213 (54.1%) | T2DM subjects: 1109 MCI with T2DM: 690 Dementia with T2DM: 132 Cognitive intact with T2DM: 287 | / | / | Diagnosis of MCI: Petersen’s diagnostic standard (memory complaint, objective memory impairment, preserved general cognitive function, minimal daily life impairment, no dementia). Diagnosis of Dementia: DSM-IIIR criteria (cognitive deficits on neuropsychological battery, impairment in social/occupational function). | OR for MCI with T2DM Risk Factors (95% CI): Current smoking: 1.36 (1.08–1.71) DM duration: 1.33 (1.07–1.59) FPG: 1.18 (1.02–1.35) HbA1c: 1.25 (1.09–1.43) Immunoreactive insulin (IRI): 1.33 (1.15–1.53) | Age, sex, education level, and other health status variables (e.g., smoking, waist circumference, BP, lipid levels). |
| Wang2017^[58]^ | cross-sectional study | China | Patients with DR and healthy controls from hospitals in China | DR group: 54.9 ± 9.9 years Control group: 54.8 ± 5.7 years | 9.48 ± 5.01 years (DR group only) | / | DR group: 8.43 ± 1.71 Control group: 5.62 ± 0.94 | Total: 37 participants (17 men, 20 women) DR group: 21 (10 men, 11 women) Control group: 16 (7 men, 9 women) | DR group (N): 21 Control group (C): 16 | / | Proliferative DR: Pre-retinal hemorrhage, vitreous hemorrhage, and neo-vascularization in the fundus. Non-proliferative DR: Microaneurysms, hard exudates, and retinal hemorrhages. Diagnostic Tools: Fundus examination, Snellen’s chart for visual acuity, and applanation tonometry for intraocular pressure. | MoCA、MMSE | Significant negative correlation between MoCA scores and ALFF values in the right occipital lobe of DR patients (p<0.01). Significant positive correlation between HbA1c levels and ALFF values in the right precuneus and lingual gyrus (p<0.01). | Age, sex, education level, fasting glucose levels, HbA1c, HOMA-IR (homeostasis model assessment method-insulin resistance), and neuropsychological test scores (MMSE, MoCA). |
| Mukherjee2021^[59]^ | cross-sectional study | India | Middle-aged patients with T2DM attending the retina clinic at Calcutta National Medical College, Kolkata, India. | DR group: 55.0 (45.0–64.0) years NODR group: 53.5 (45.0–64.0) years | DR group: 6.0 (2.0–10.0) years NODR group: 5.0 (1.0–10.0) years | / | DR group: 7.1 ± 1.9 NODR group: 6.8 ± 1.0 | DR group: 36 (19 men, 17 women) NODR group: 36 (16 men, 20 women) | DR group (N): 36 NODR group (C): 36 | / | Proliferative DR: Pre-retinal hemorrhage, vitreous hemorrhage, and neo-vascularization. Non-proliferative DR: Microaneurysms, hard exudates, and retinal hemorrhages. Diagnostic Tools: Dilated fundoscopy graded per modified ETDRS guidelines. | MMSE: Assessed 5 domains (orientation, registration, attention/calculation, recall, language). Cognitive impairment defined as: Dementia: 0–22 Mild cognitive impairment: 23–26 Normal cognition: 27–30 Education-adjusted MMSE scores were also used. | Overall DR vs. NODR: OR 4.8 (95% CI 1.02–22.68) for education-adjusted cognitive impairment. By DR Severity: Mild NPDR: OR 1.77 (95% CI 0.28–11.08) Moderate NPDR: OR 4.34 (95% CI 1.12–16.76) Advanced DR: OR 9.30 (95% CI 1.92–45.10 | Age, duration of diabetes, BMI, urine microalbumin:creatinine ratio (ACR), HbA1c, education level, and sex. |
| Zhu2024^[60]^ | cross-sectional study | China | T2DM patients treated at the Affiliated Hospital 6 of Nantong University and The Third Affiliated Hospital of Soochow University | DR group: 57.56 ± 4.39 years Simple diabetes group: 56.25 ± 7.39 years | DR group: 5.22 ± 1.36 years Simple diabetes group: 5.37 ± 1.16 years | / | DR group: 11.42 ± 1.56 Simple diabetes group: 8.07 ± 1.75 | 80 patients (DR group: 26 males out of 46; Simple diabetes group: 21 males out of 34). | DR group (n=46), Simple diabetes group (n=34). | / | Diagnosed using FFA based on specific retinal changes (microvascular tumors, bleeding points, exudates, etc.). | MMSE, TMT-A and TMT-B. | Correlations were reported (e.g., RNFLT negatively correlated with TMT-A and TMT-B, positively correlated with MMSE). | Blood pressure (SBP, DBP), BMI, blood lipids (triglycerides, cholesterol, LDL, HDL), FINS, HOMA-IR index, ApoB/ApoA1. |
| Zheng2014^[61]^ | cross-sectional study | China | Hospitalized patients with T2DM from the Metabolic Disease Hospital of Tianjin Medical University | 74.36 ± 4.54 (control group), 75.16 ± 4.65 (MCI group) | Median (inter-quartile range): 17.0 (10.0, 22.0) years (control group), 15.0 (9.05, 20.0) years (MCI group) | / | 8.42 ± 2.08 (control group), 8.89 ± 2.45 (MCI group) | 100 patients (34% male in both groups) | 50/50 | / | DR was obtained from medical records; no specific diagnostic tool mentioned. | MMSE and a short memory questionnaire. | N/A | Duration of diabetes, GHbA1c, diabetic retinopathy, BMI, smoking status, systolic blood pressure, triglycerides, and total cholesterol. |
| Roy2015^[62]^ | cross-sectional study | United States | Patients from an internal medicine office at Cooper University Hospital, New Jersey | Mean age 50 ± 9 years (range: 26–60 years) | <5 years: 17.1% 5–10 years: 54.9% 10 years: 28.0% | / | Optimal control (<7%): 11.6% with cognitive impairment Suboptimal control (≥7%): 30.2% with cognitive impairment | 82 (53.7%) | Normal cognitive function (3MS score ≥79): 66 patients Cognitive impairment (3MS score <79): 16 patients | / | DR was identified via medical records. Prevalence: 9.8% overall (31.2% in cognitive impairment group vs. 4.5% in normal group). | Cognitive impairment defined as 3MS score <79. | Higher DR prevalence in cognitive impairment group (31.2% vs. 4.5%; P < 0.001). Weak negative correlation between HbA1c and cognitive function (*r* = −0.292). | Age, gender, race, education level, duration of diabetes, comorbid conditions (e.g., HTN, depression, CKD), and management modality (diet, oral hypoglycemics, insulin). |
| Ogama2019^[63]^ | cross-sectional study | Japan | Outpatients with T2DM treated at the National Center for Geriatrics and Gerontology (NCGG) hospital | Total: 75.0 ± 5.3 years Cognitive impairment group: 76.0 ± 5.8 years Normal cognition group: 74.2 ± 4.7 years | Total: 13.3 ± 10.8 years Cognitive impairment group: 13.3 ± 10.6 years Normal cognition group: 13.4 ± 11.0 years | / | Total: 7.1 ± 0.6 Cognitive impairment group: 7.3 ± 0.7 Normal cognition group: 7.0 ± 0.5 | 69 (52.2%) | Cognitive impairment group (n=32), Normal cognition group (n=37). | / | DR was fundoscopically assessed through dilated pupils by experienced ophthalmologists. | MMSE | The prevalence of sarcopenia was compared between groups (21.9% in cognitive impairment vs. 2.7% in normal cognition, p=0.021). | Age, HbA1c level, presence of diabetic neuropathy, blood lipids (TG, TC, HDL, LDL), estimated eGFR, serum albumin, and UACR. |
| Murata2017^[64]^ | cross-sectional study | Japan | Elderly Japanese patients withT2DM visiting the outpatient clinic of the Diabetic Center, Fuchu Hospital | 73.4 ± 6.1 | 17.4 ± 10.0 | / | 7.6 ± 1.2 | 281(51.2% ) | Not explicitly divided into groups; cognitive function was categorized as normal (MMSE 27–30), mild cognitive impairment (MCI, MMSE 24–26), and dementia (MMSE ≤23). | / | Pre- or proliferative DR (P-Retinopathy) was assessed, but the specific diagnostic tool was not detailed. | MMSE | Prevalence comparisons were made (e.g., 33.3% in dementia range vs. 18.4% in normal range). | Age, schooling history, fasting serum NEFA, eGFR, insulin treatment, HTN, TC, HbA1c, and DM duration. |
| Blanquisco2017^[65]^ | cross-sectional study | Philippines | General Medicine and Diabetes clinics of the Philippine General Hospital (PGH) | 67±4.8 | Median 12 years (IQR ±12) | / | 7.6±1.9 | 133 (30.1%) | Without MCI: 73 With MCI: 60 | / | Funduscopic examination via dilated pupils by an ophthalmologist, classified according to the ETDRS and International Clinical Diabetic Retinopathy Disease Severity Scales | MoCA-P, cutoff score ≤21 | Unadjusted OR: 1.45 (0.68, 3.11), p-value 0.338 | Age, sex, civil status, education, duration of diabetes, treatment (insulin, OHA), comorbidities ( HTN, dyslipidemia, IHD, HF, CKD), smoking, hypoglycemia, BMI, abdominal obesity, BP, lipid profile (HDL, non-HDL), and other microvascular complications (neuropathy, DN). |
| Crosby-nwaobi2013^[66]^ | cross-sectional study | United Kingdom | Population-based eye screening program in South East London | 64.8 ± 10.8 | 17.7 ± 8.4 | / | 8.3 ± 1.9 | 380 (56.2%) | No/mild DR (N): 252 Proliferative DR (C): 128 | / | Graded using retinal eye photographs according to Early Treatment of Diabetic Retinopathy Study criteria. No/mild retinopathy ≤ level 35; proliferative DR ≥ level 61. | ACE-R, MMSE, Mini-Cog | The no/mild DR group had lower cognitive scores (adjusted mean ± SE: 77.0 ± 1.9) compared to the PDR group (82.5 ± 2.2,P<0.001). MMSE cutoff scores showed 12% of the no/mild DR group screened positive for dementia/cognitive impairment vs. 5% in the PDR group. | Age, sex, ethnicity, education, visual acuity, DM duration, socioeconomic status, nephropathy, BMI, DBP, HbA1c, TG, TC, alcohol consumption, severe emotional distress, and depressive symptomatology. |
| Xia2020^[67]^ | cross-sectional study | China | Inpatients from the Department of Endocrinology, Tongji Hospital, Tongji Medical College, Huazhong University of Science and Technology | 56.8 ± 6.9 | 8.7 ± 6.7 | 48.1 ± 8.7 | 8.9 ± 2.1 | 297 (64.6%) | Dementia group: 47 Non-dementia group: 250 (further divided into MCI: 174 and normal cognition: 76) | / | DR was assessed as part of diabetic complication screening. | CDR, MMSE, MOCA | For dementia: OR 2.197 (95% CI: 1.035–4.664) For MCI: Not significant (DR unrelated to MCI) | Age, sex, and education level |
| Ding2010^[68]^ | cross-sectional study | United Kingdom | Population-based cohort from the Lothian Diabetes Register, Scotland | 67.3 ± 4.2 years (no DR), 67.4 ± 4.2 years (mild DR), 67.1 ± 4.2 years (moderate-to-severe DR) | Median (IQR): 5.5 (3.4–9.4) years (no DR), 9.3 (5.1–14.4) years (mild DR), 17.1 (12.1–22.9) years (moderate-to-severe DR) | / | 7.3 ± 1.1 (no DR), 7.5 ± 1.1 (mild DR), 8.4 ± 1.4 (moderate-to-severe DR) | 1,044 participants (49.2% male in no DR group, 55.1% in mild DR, 57.4% in moderate-to-severe DR) | 705 (no DR), 292 (mild DR), 47 (moderate-to-severe DR) | / | Graded using standard seven-field binocular digital retina photography and the ETDRS scale. DR severity: none (ETDRS level <20), mild (levels 20–35), moderate-to-severe (levels ≥43). | Battery of seven tests (e.g., Wechsler Memory Scale-III, Wechsler Adult Intelligence Scale, Trail Making Test) and general cognitive ability score derived from principal component analysis. MHVS estimated premorbid ability. | In men, moderate-to-severe DR was associated with worse g (P<0.001), verbal fluency (P=0.001), and processing speed (P=0.001) after full adjustment. No significant associations in women. | Age, sex, MHVS (premorbid ability), education, alcohol intake, smoking, waist-to-hip ratio, SBP, TC, macrovascular disease, and depression symptoms. Notably, DM duration and HbA1c were excluded from the main model to avoid overadjustment. |

ACD: All-cause Dementia; ACE-R: Addenbrooke’s Cognitive Examination-Revised; AD: Alzheimer’s Disease; BDR: Background Diabetic Retinopathy; BMI: Body Mass Index; BP: Blood Pressure; CASI: Cognitive Abilities Screening Instrument; CDR: Clinical Dementia Rating; CHD: Coronary Heart Disease; CKD: Chronic Kidney Disease; CVD: Cardiovascular Disease; DBP: Diastolic Blood Pressure; DN: Diabetic Nephropathy; DR: Diabetic Retinopathy; eGFR: Estimated Glomerular Filtration Rate; EMR: Electronic Medical Records; ETDRS: Early Treatment Diabetic Retinopathy Study; FFA: Fundus Fluorescein Angiography; FPG: Fasting Plasma Glucose; GCS: Global Cognitive Score; GCS(z): Global Cognitive Score (z-score); GDS-15: Geriatric Depression Scale 15; HbA1c: Glycated Hemoglobin; HDL: High-Density Lipoprotein; HF: Heart Failure; HTN: Hypertension; IHD: Ischemic Heart Disease; LDL: Low-Density Lipoprotein; MHVS: Mill-Hill Vocabulary Scale; MMSE: Mini-Mental State Examination; MoCA: Montreal Cognitive Assessment; NEFA: Non-esterified Fatty Acid; NPDR: Non-Proliferative Diabetic Retinopathy; PA: Physical Activity; PDR: Proliferative Diabetic Retinopathy; PHQ-9: Patient Health Questionnaire-9; PPDR: Pre-Proliferative Diabetic Retinopathy; RBI: Regression-Based Index; RVMs: Retinal Vascular Measures; SBP: Systolic Blood Pressure; SIVA: Singapore I Vessel Assessment; TC: Total Cholesterol; TG: Triglycerides; TICSM: Modified Telephone Interview for Cognitive Status; TMT: Trail Making Test; T2DM: Type 2 Diabetes Mellitus; UACR: Urinary Albumin-to-Creatinine Ratio; VD: Vascular Dementia; WMLs: White Matter Lesions; 6-CIT: 6-Item Cognitive Impairment Test; 3MS: Modified Mini-Mental State Examination.

**Table S2. The quality of cross-sectional studies.**

| **Item** | Liu2016 | Liu2017 | Su2021 | Wu2022 | Xiao2023 | Yang2024 | Zhu2019 | Finger2014 | Moran2016 | Sanahuja2016 | Gorska2014 | Gorska2016 | Yu ZW 2020 | Sajeev2023 | Yokoyama2014 | Tekin2009 | Tong2014 | Gao2016 | Wang2017 | Mukherjee2021 | Zhu2024 | Zheng2014 | Roy2015 | Ogama2019 | Murata2017 | Blanquisco2017 | Crosby-nwaobi 2013 | Xia 2020 | Ding2010 |
| --- | --- | --- | --- | --- | --- | --- | --- | --- | --- | --- | --- | --- | --- | --- | --- | --- | --- | --- | --- | --- | --- | --- | --- | --- | --- | --- | --- | --- | --- |
| 1) Define the source of information (survey, record review) | yes | yes | yes | yes | yes | yes | yes | yes | yes | yes | yes | yes | yes | yes | yes | yes | yes | yes | yes | yes | yes | yes | yes | yes | yes | yes | yes | yes | yes |
| 2) List inclusion and exclusion criteria for exposed and unexposed subjects (cases and controls) or refer to previous publications | yes | yes | yes | yes | yes | yes | yes | unclear | yes | yes | yes | yes | yes | yes | yes | yes | yes | yes | yes | yes | yes | yes | yes | yes | yes | yes | yes | yes | yes |
| 3) Indicate time period used for identifying patients | yes | unclear | yes | yes | yes | yes | yes | yes | yes | unclear | unclear | unclear | yes | yes | yes | yes | unclear | yes | unclear | yes | yes | yes | yes | yes | unclear | yes | no | yes | unclear |
| 4) Indicate whether or not subjects were consecutive if not population-based | unclear | unclear | unclear | unclear | unclear | unclear | unclear | unclear | unclear | unclear | unclear | unclear | unclear | unclear | yes | yes | unclear | unclear | unclear | yes | unclear | unclear | yes | unclear | unclear | yes | yes | yes | yes |
| 5) Indicate if evaluators of subjective components of study were masked to other aspects of the status of the participant | no | no | yes | no | no | no | no | no | yes | yes | unclear | unclear | unclear | unclear | unclear | yes | yes | unclear | no | yes | unclear | unclear | unclear | unclear | unclear | unclear | unclear | unclear | unclear |
| 6) Describe any assessments undertaken for quality assurance purposes (e.g., test/retest of primary outcome measurements) | unclear | unclear | yes | unclear | unclear | unclear | unclear | unclear | unclear | unclear | unclear | unclear | unclear | unclear | unclear | unclear | unclear | unclear | unclear | unclear | unclear | unclear | no | no | no | yes | yes | yes | yes |
| 7) Explain any patient exclusions from analysis | unclear | yes | yes | yes | yes | yes | yes | yes | yes | yes | yes | yes | yes | unclear | unclear | yes | yes | yes | yes | unclear | unclear | unclear | unclear | unclear | unclear | yes | yes | yes | yes |
| 8) Describe how confounding was assessed and/or controlled. | no | yes | yes | unclear | yes | yes | yes | yes | yes | yes | yes | yes | yes | unclear | yes | yes | yes | yes | unclear | yes | unclear | yes | unclear | unclear | yes | yes | yes | yes | yes |
| 9) If applicable, explain how missing data were handled in the analysis | unclear | unclear | unclear | no | no | no | no | unclear | no | unclear | unclear | unclear | unclear | no | no | no | no | no | unclear | unclear | unclear | unclear | no | no | no | no | no | no | no |
| 10) Summarize patient response rates and completeness of data collection | unclear | unclear | unclear | unclear | unclear | yes | yes | unclear | yes | unclear | unclear | unclear | unclear | unclear | unclear | unclear | unclear | unclear | unclear | unclear | unclear | unclear | unclear | unclear | unclear | yes | yes | yes | unclear |
| 11) Clarify what follow-up, if any, was expected and the percentage of patients for which incomplete data or follow-up was obtained | unclear | unclear | unclear | unclear | unclear | unclear | unclear | unclear | unclear | unclear | unclear | unclear | unclear | unclear | unclear | unclear | unclear | unclear | unclear | unclear | unclear | unclear | unclear | unclear | unclear | unclear | unclear | unclear | unclear |
| [Quality](javascript:;) [assessment](javascript:;) | moderate | moderate | moderate | moderate | moderate | moderate | moderate | moderate | moderate | moderate | moderate | moderate | moderate | moderate | moderate | moderate | moderate | moderate | moderate | moderate | moderate | moderate | moderate | moderate | moderate | moderate | moderate | moderate | moderate |

**Table S3. The quality of case-control studies or cohort studies.**

| **Study** | **Selection** | **Comparability** | **Exposure/Outcome** | **[Quality](javascript:;) [assessment](javascript:;)** |
| --- | --- | --- | --- | --- |
| Case-control | | | | |
| Oğuz 2009 | ★★★★ | ★★ | ★★ | high |
| Naidu 2016 | ★★★ | ★★ | ★★ | high |
| Lu2019 | ★★★★ | ★★ | ★★ | high |
| Maimaitituerxun2024 | ★★★★ | ★★ | ★★ | high |
| Cohort | | | | |
| Umegaki2012 | ★★★ | ★★ | ★★★ | high |
| Bruce2014 | ★★★ | ★★ | ★★★ | high |
| Trento2017 | ★★★ | ★★ | ★★★ | high |
| de Bresser2010 | ★★★ | ★★ | ★★★ | high |
| Yen2024 | ★★★★ | ★★ | ★★★ | high |
| Ravazzani2022 | ★★★★ | ★★ | ★ | high |
| Tlemçani2022 | ★★★★ | ★★ | ★ | high |
| Doney2022 | ★★★★ | ★★ | ★★ | high |
| Lee2023 | ★★★★ | ★★ | ★★★ | high |
| Roberts2014 | ★★★★ | ★★ | ★★★ | high |
| Hendrie2018 | ★★★★ | ★★ | ★★★ | high |
| Yu Ji Hee2020 | ★★★★ | ★★ | ★★★ | high |
| Hugenschmidt2014 | ★★★★ | ★★ | ★★★ | high |
| Exalto2014 | ★★★★ | ★★ | ★★★ | high |
| Zhong2023 | ★★★ | ★★ | ★★★ | high |

**Table S4. Sensitivity analysis – comparison of DL vs. REML models for meta-analysis of overall cognitive impairment by study type**

| study type | DL (OR, 95% CI, P) | REML (OR, 95% CI, P) |
| --- | --- | --- |
| cross-sectional study | 2.039 (1.722–2.416), <0.001 | 2.039 (1.722–2.416), <0.001 |
| cohort study | 1.126 (1.009–1.256), 0.034 | 1.191 (0.973–1.458), 0.089 |

"Abbreviations: DL, DerSimonian-Laird random-effects model; REML, restricted maximum likelihood random-effects model; OR, odds ratio; CI, confidence interval.

**Table S5. Sensitivity analysis comparing DL and REML models: Meta-analysis results for cognitive impairment subtypes by study design**

| subgroup | study type | DL (OR, 95% CI, P) | REML (OR, 95% CI, P) |
| --- | --- | --- | --- |
| Cognitive decline | cross-sectional study | 1.750 (1.252–2.446), 0.001 | 1.750 (1.252–2.446), 0.001 |
|  | cohort study | 1.117 (0.840–1.484), 0.448 | 1.168 (0.808–1.687), 0.409 |
| MCI | cross-sectional study | 2.146 (1.751–2.630), <0.001 | 2.146 (1.751–2.630),<0.001 |
|  | cohort study | 0.572 (0.055–6.006), 0.642 | 0.572 (0.055–6.006), 0.642 |
| Dementia | cross-sectional study | 2.200 (1.036–4.670), 0.040 | 2.200 (1.036–4.670), 0.040 |
|  | cohort study | 1.166 (1.055–1.288), 0.003 | 1.190 (1.037–1.365), 0.013 |
| AD | cohort study | 1.200 (1.027–1.402), 0.022 | 1.199 (1.027–1.400), 0.021 |
| VD | cohort study | 1.112 (1.026–1.206), 0.010 | 1.115 (1.020–1.220), 0.017 |
| Overall | cross-sectional study | 1.754 (1.198–2.568), 0.004 | 1.716 (1.301–2.262), <0.001 |
|  | cohort study | 1.115 (1.059–1.174), <0.001 | 1.156 (1.040–1.284), 0.007 |

a Random-effects models were used for all pooled estimates; REML was employed as a sensitivity analysis. b Subgroups without cross-sectional studies (AD, VD) are presented with cohort data only."

**Table S6. Sensitivity analysis comparing DL and REML models: Subgroup analysis for cognitive impairment by duration of diabetes**

| subgroup | study type | DL (OR, 95% CI, P) | REML (OR, 95% CI, P) |
| --- | --- | --- | --- |
| ＜10 years | cross-sectional study | 2.341 (1.533–3.572), <0.001 | 2.367 (1.518–3.690),<0.001 |
|  | cohort study | 1.079 (0.879–1.325), 0.467 | 1.093 (0.847–1.412), 0.494 |
| ≥10 years | cross-sectional study | 2.046 (1.595–2.625), <0.001 | 2.046 (1.595–2.625), <0.001 |
|  | cohort study | 1.258 (0.548–2.890), 0.588 | 1.198 (0.357–4.022), 0.770 |
| Overall | cross-sectional study | 2.111 (1.724–2.585), <0.001 | 2.116 (1.792–2.500), <0.001 |
|  | cohort study | 1.150 (0.953–1.288), 0.145 | 1.175 (0.874–1.579), 0.286 |

**Table S7. Sensitivity analysis comparing DL and REML models: Subgroup analysis for cognitive impairment by follow-up duration**

| subgroup | DL (OR, 95% CI, P) | REML (OR, 95% CI, P) |
| --- | --- | --- |
| ＜10 years | 1.068 (0.957–1.192), 0.242 | 1.082 (0.890–1.316), 0.429 |
| ≥10 years | 1.906 (1.237–2.935), 0.003 | 1.935 (1.224–3.060), 0.005 |
| Overall | 1.125 (1.008–1.255), 0.036 | 1.189 (0.968–1.461), 0.098 |

**Table S8. Sensitivity analysis comparing DL and REML models: Subgroup analysis for cognitive impairment by cognitive assessment tools**

| subgroup | study type | DL (OR, 95% CI, P) | REML (OR, 95% CI, P) |
| --- | --- | --- | --- |
| MMSE | cross-sectional study | 1.820 (1.039–3.187), 0.036 | 1.820 (1.039–3.187), 0.036 |
|  | cohort study | 1.052 (0.697–1.587), 0.810 | 1.091 (0.504–2.364), 0.825 |
| MoCA | cross-sectional study | 2.146 (1.751–2.630), <0.001 | 2.146 (1.751–2.630),<0.001 |
| Overall | cross-sectional study | 2.105 (1.739–2.549), <0.001 | 2.105 (1.739–2.549), <0.001 |
|  | cohort study | 1.052 (0.697–1.587), 0.810 | 1.091 (0.504–2.364), 0.825 |

**Table S9. The pooled effect size by trim-and fill method in cohort studies.**

| **Studies** | **logOR** | **95%Cl** |
| --- | --- | --- |
| **Observed** | 0.175 | -0.027, 0.377 |
| **Observed + Imputed** | 0.111 | -0.109, 0.331 |

**Table S10. PRISMA Checklist**

| Section and Topic | Item # | **PRISMA Checklist item** | Location where item is reported |
| --- | --- | --- | --- |
| TITLE | | |  |
| Title | 1 | Identify the report as a systematic review. | 1 |
| ABSTRACT | | |  |
| Abstract | 2 | See the PRISMA 2020 for Abstracts checklist. (made as per the Journal guidelines) | 2 |
| INTRODUCTION | | |  |
| Rationale | 3 | Describe the rationale for the review in the context of existing knowledge. | 3 |
| Objectives | 4 | Provide an explicit statement of the objective(s) or question(s) the review addresses. | 3 |
| METHODS | | |  |
| Eligibility criteria | 5 | Specify the inclusion and exclusion criteria for the review and how studies were grouped for the syntheses. | 4 |
| Information sources | 6 | Specify all databases, registers, websites, organisations, reference lists and other sources searched or consulted to identify studies. Specify the date when each source was last searched or consulted. | 4; Supplementary methods |
| Search strategy | 7 | Present the full search strategies for all databases, registers and websites, including any filters and limits used. | 4; Supplementary methods |
| Selection process | 8 | Specify the methods used to decide whether a study met the inclusion criteria of the review, including how many reviewers screened each record and each report retrieved, whether they worked independently, and if applicable, details of automation tools used in the process. | 5 |
| Data collection process | 9 | Specify the methods used to collect data from reports, including how many reviewers collected data from each report, whether they worked independently, any processes for obtaining or confirming data from study investigators, and if applicable, details of automation tools used in the process. | 5,6; Figure 1 |
| Data items | 10a | List and define all outcomes for which data were sought. Specify whether all results that were compatible with each outcome domain in each study were sought (e.g., for all measures, time points, analyses), and if not, the methods used to decide which results to collect. | 5; Tables S1 |
|  | 10b | List and define all other variables for which data were sought (e.g., participant and intervention characteristics, funding sources). Describe any assumptions made about any missing or unclear information. | 5;Tables S1 |
| Study risk of bias assessment | 11 | Specify the methods used to assess risk of bias in the included studies, including details of the tool(s) used, how many reviewers assessed each study and whether they worked independently, and if applicable, details of automation tools used in the process. | 8,9; Tables S2, S3 |
| Effect measures | 12 | Specify for each outcome the effect measure(s) (e.g. risk ratio, mean difference) used in the synthesis or presentation of results. | 5,6 |
| Synthesis methods | 13a | Describe the processes used to decide which studies were eligible for each synthesis (e.g. tabulating the study intervention characteristics and comparing against the planned groups for each synthesis (item #5)). | 5,6 |
|  | 13b | Describe any methods required to prepare the data for presentation or synthesis, such as handling of missing summary statistics, or data conversions. | 5,6 |
|  | 13c | Describe any methods used to tabulate or visually display results of individual studies and syntheses. | 5,6 |
|  | 13d | Describe any methods used to synthesize results and provide a rationale for the choice(s). If meta-analysis was performed, describe the model(s), method(s) to identify the presence and extent of statistical heterogeneity, and software package(s) used. | 6 |
|  | 13e | Describe any methods used to explore possible causes of heterogeneity among study results (e.g. subgroup analysis, meta-regression). | 6 |
|  | 13f | Describe any sensitivity analyses conducted to assess robustness of the synthesized results. | 6 |
| Reporting bias assessment | 14 | Describe any methods used to assess risk of bias due to missing results in a synthesis (arising from reporting biases). | 6 |
| Certainty assessment | 15 | Describe any methods used to assess certainty (or confidence) in the body of evidence for an outcome. | 6 |
| RESULTS | | |  |
| Study selection | 16a | Describe the results of the search and selection process, from the number of records identified in the search to the number of studies included in the review, ideally using a flow diagram. | 6, Figure 1 |
|  | 16b | Cite studies that might appear to meet the inclusion criteria, but which were excluded, and explain why they were excluded. | 6, Figure 1 |
| Study characteristics | 17 | Cite each included study and present its characteristics. | 8, Table S1 |
| Risk of bias in studies | 18 | Present assessments of risk of bias for each included study. | 8, 9; Tables S2, S3 |
| Results of individual studies | 19 | For all outcomes, present, for each study: (a) summary statistics for each group (where appropriate) and (b) an effect estimate and its precision (e.g. confidence/credible interval), ideally using structured tables or plots. | Table S1 |
| Results of syntheses | 20a | For each synthesis, briefly summarise the characteristics and risk of bias among contributing studies. | 9–14; Figures 2–5, S1 |
|  | 20b | Present results of all statistical syntheses conducted. If meta-analysis was done, present for each the summary estimate and its precision (e.g. confidence/credible interval) and measures of statistical heterogeneity. If comparing groups, describe the direction of the effect. | 9–14; Figures 2–5, S1 |
|  | 20c | Present results of all investigations of possible causes of heterogeneity among study results. | 12–14; Figures 4, 5, S1 |
|  | 20d | Present results of all sensitivity analyses conducted to assess the robustness of the synthesized results. | 14; Figures S1–S4, Tables S4–S8 |
| Reporting biases | 21 | Present assessments of risk of bias due to missing results (arising from reporting biases) for each synthesis assessed. | 14–15; Figures S5–S6, Table S9 |
| Certainty of evidence | 22 | Present assessments of certainty (or confidence) in the body of evidence for each outcome assessed. | 14; Figures S1–S4 |
| DISCUSSION | | |  |
| Discussion | 23a | Provide a general interpretation of the results in the context of other evidence. | 15-18 |
|  | 23b | Discuss any limitations of the evidence included in the review. | 18 |
|  | 23c | Discuss any limitations of the review processes used. | 18 |
|  | 23d | Discuss implications of the results for practice, policy, and future research. | 16,17 |
| OTHER INFORMATION | | |  |
| Registration and protocol | 24a | Provide registration information for the review, including register name and registration number, or state that the review was not registered. | 4 |
|  | 24b | Indicate where the review protocol can be accessed, or state that a protocol was not prepared. | 4 |
|  | 24c | Describe and explain any amendments to information provided at registration or in the protocol. | NA |
| Support | 25 | Describe sources of financial or non-financial support for the review, and the role of the funders or sponsors in the review. | 19 |
| Competing interests | 26 | Declare any competing interests of review authors. | 19 |
| Availability of data, code and other materials | 27 | Report which of the following are publicly available and where they can be found: template data collection forms; data extracted from included studies; data used for all analyses; analytic code; any other materials used in the review. | 19 |

**
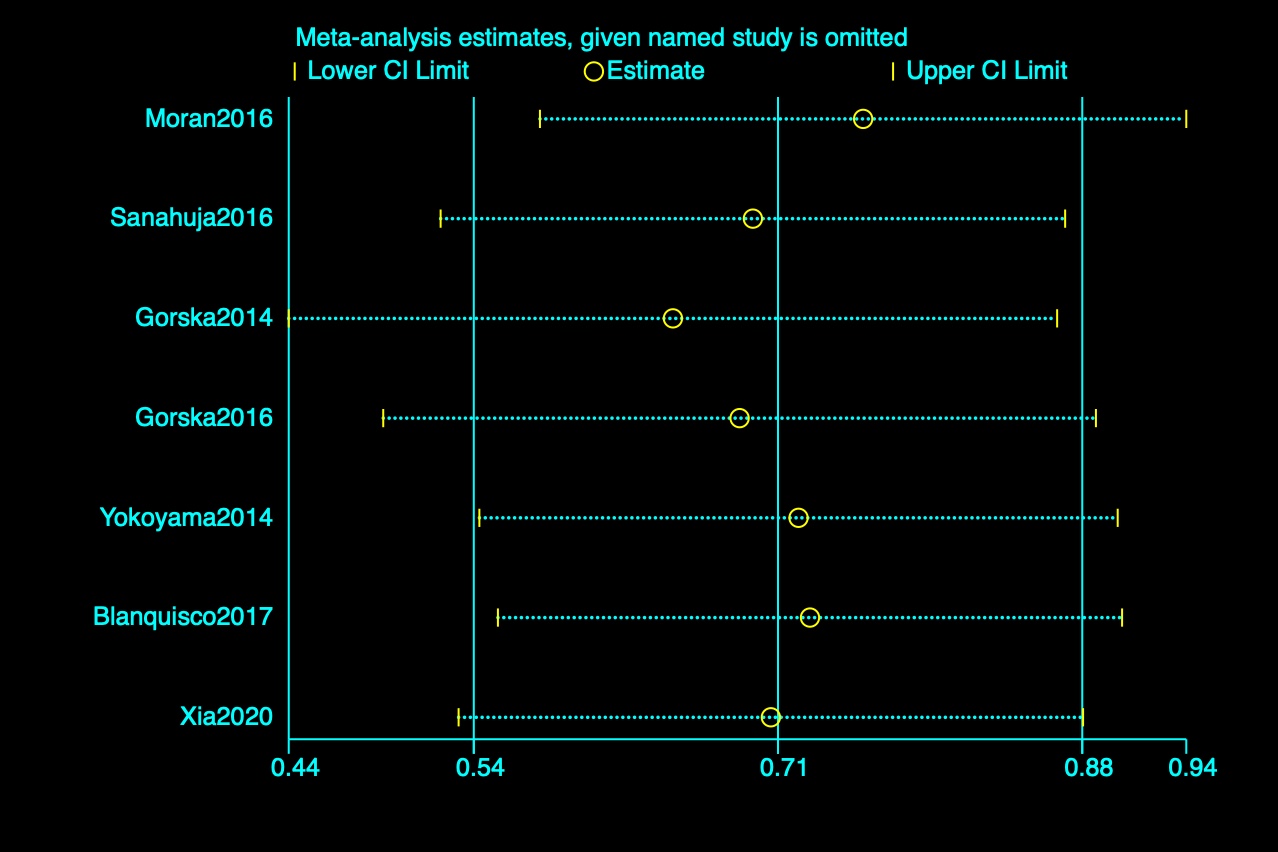
**

**Figure S1. Sensitivity analysis results for cross-sectional studies (fix model).**

**
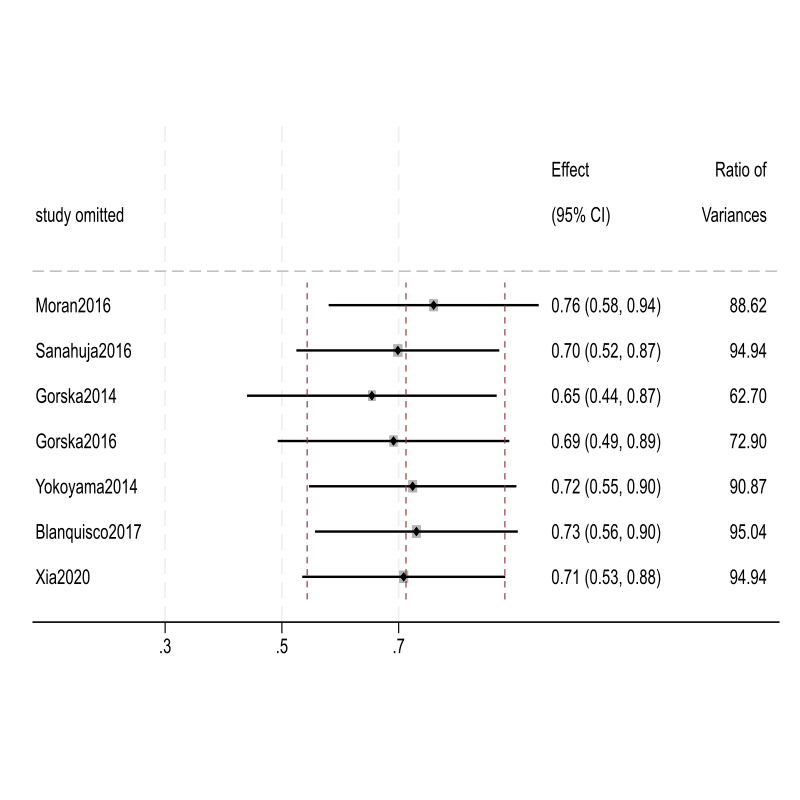
**

**Figure S2. Leave-one-out sensitivity analysis for cross-sectional studies.**

**
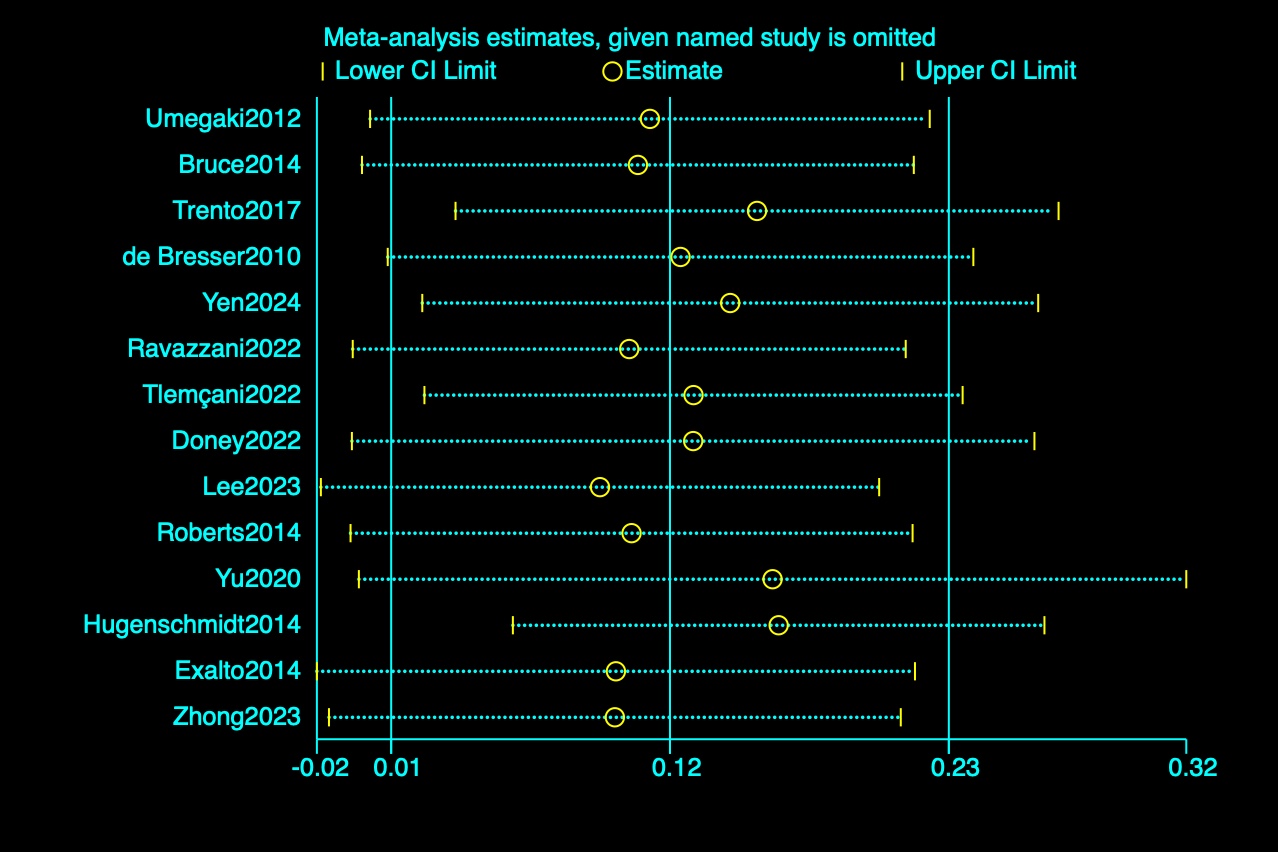
**

**Figure S3. Sensitivity analysis results for cohort studies (random model).**

**
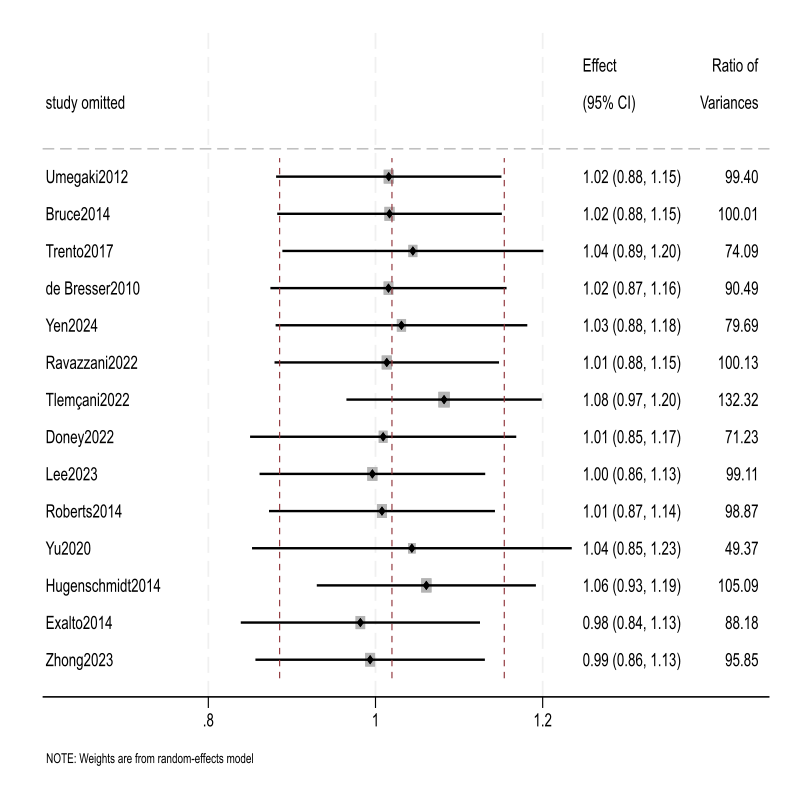
**

**Figure S4. Leave-one-out sensitivity analysis for cohort studies.**


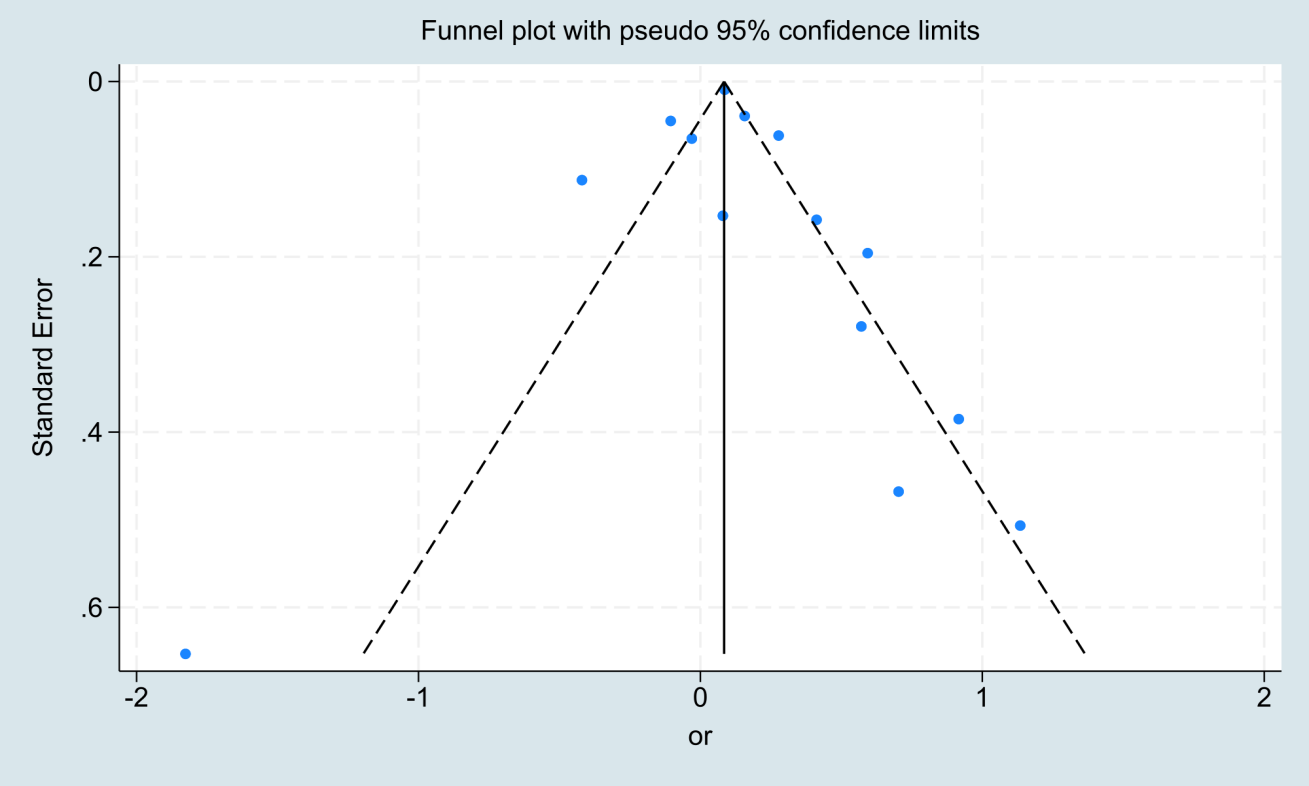


**Figure S5.** Funnel plot of standard error of log OR for the association of diabetic retinopathy and cognitive impairment.


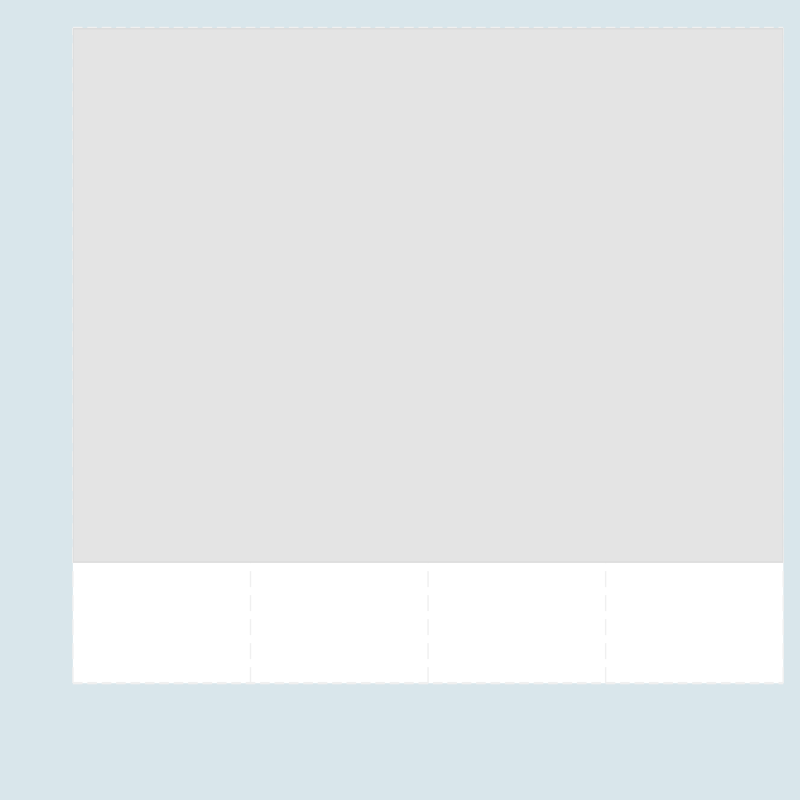


**Figure S6.** Enhanced funnel plot of standard error of log OR for the association of diabetic retinopathy and cognitive impairment.
